# Intrinsic theta oscillation in the attractor network of grid cells

**DOI:** 10.1016/j.isci.2023.106351

**Published:** 2023-03-14

**Authors:** Ziqun Wang, Tao Wang, Fan Yang, Feng Liu, Wei Wang

**Affiliations:** 1 National Laboratory of Solid State Microstructures, Department of Physics, Collaborative Innovation Center of Advanced Microstructures, and Institute for Brain Sciences, Nanjing University, Nanjing 210093, P. R. China

**Keywords:** Cell, Neuroscience

## Abstract

Both grid-like firing fields and theta oscillation are hallmarks of grid cells in the mammalian brain. While bump attractor dynamics have generally been recognized as the substrate for grid firing fields, how theta oscillation arises and interacts with persistent activity in a cortical circuit remains obscure. Here, we report that the theta oscillation intrinsically emerges in a continuous attractor network composed of principal neurons and interneurons. Periodic bump attractors and the theta rhythm stably coexist in both cell types due to the division of labor among interneurons via structured synaptic connectivity between principal cells and interneurons. The slow dynamics of NMDAR-mediated synaptic currents support the persistency of bump attractors and restrict the oscillation frequency in the theta band. The spikes of neurons within bump attractors are phase locked to a proxy of local field potential. The current work provides a network-level mechanism that orchestrates the bump attractor dynamics and theta rhythmicity.

## Highlights

• Theta rhythm emerges intrinsically in a continuous attractor network (CAN) of grid cells
• Labor division among interneurons ensures both the persistent and rhythmic activity
• Dominance and slow dynamics of NMDA receptors are required at recurrent synapses
• Neural firings are phase locked to the local field potential (LFP) and external rhythmic input

## Graphical abstract

**Figure:**
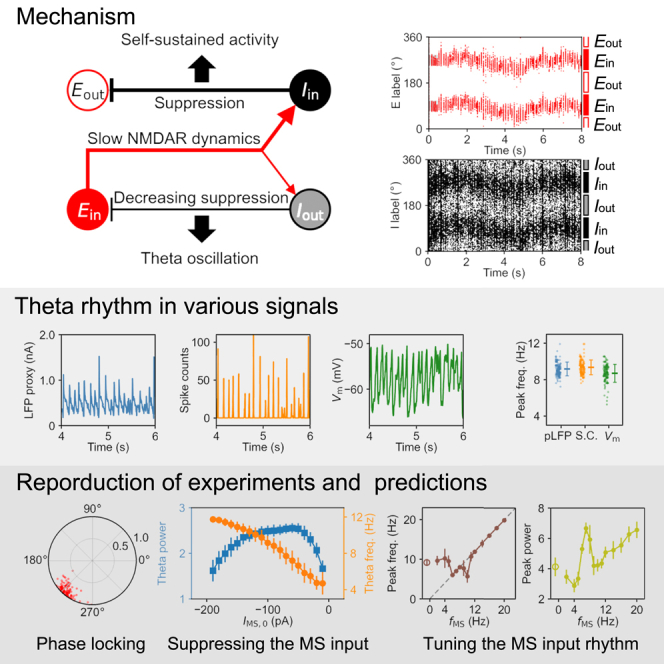

## Introduction

Grid cells in the medial entorhinal cortex (mEC) constitute a major cell type in the brain’s navigation system; their hexagonally distributed firing fields provide a spatial metric.^1^^,^^2^ Their temporal characteristic, typically theta oscillation (4–12 Hz), is another important aspect of functioning.^3^ This theta rhythm is present in spike trains of individual grid cells and the local field potential (LFP).^3^^,^^4^ Functionally, theta oscillation is strongly associated with the movement of animals, with its frequency possibly reflecting the speed or acceleration information.^5^^,^^6^ Sensory information received by the hippocampal-entorhinal circuit can be integrated within temporal windows provided by theta oscillation.^7^ The uncertainty about the animal’s position can be significantly reduced by taking into account the timing of neural spiking relative to the LFP theta phase.^8^ Indeed, the hexagonal lattice of firing fields and theta rhythm are closely correlated. For instance, cells with the same grid scale and orientation constitute a grid module and exhibit more coherent oscillations than intermodular ones;^9^ there is a significant negative correlation between the theta frequency and grid spacing of individual cells.^9^^,^^10^^,^^11^ When a rat moves through one firing field of a grid cell, either phase locking or phase precession appears between its spike train and the LFP, and the degree of precession depends heavily on the rat’s position.^3^^,^^12^^,^^13^^,^^14^ Thus, exploring both the features within the same framework is essential to unraveling the dynamics and function of grid cells.

Two major kinds of computational models have been proposed to account for spatiotemporal firing features of grid cells: the continuous attractor network (CAN) model and oscillatory interference (OI) model, which are at the network and single-neuron levels, respectively. With the CAN model, the hexagonally distributed activation bumps on a neural sheet with toroidal topology can be topographically mapped to the two-dimensional (2D) space where the animal moves.^15^^,^^16^^,^^17^ Moreover, given the specific boundary condition of the neural connectivity profile, the CAN model admitting a single bump attractor can also account for the grid firing pattern.^18^ With the OI model, the interference between the theta oscillation with constant frequency and that with the frequency varying with the animal’s velocity underlies the periodic firing fields of single cells.^5^^,^^11^^,^^19^ Although many experimental results can be understood in the framework of either the CAN model or the OI model, few studies tried to combine them,^20^^,^^21^ partly due to their radical differences in assumption and dynamics. Notably, both the assumptions and predictions of the CAN model and its extensions have been extensively validated in experiments.^4^^,^^12^^,^^17^^,^^22^^,^^23^

Two mechanisms underlying theta oscillation in mEC have been suggested. The theta rhythm could result from the input from the medial septum (MS). Interneurons in superficial layers of the mEC receive strong inputs from GABAergic neurons in MS, which are thought to be theta pacemakers.^24^^,^^25^^,^^26^ Alternatively, a special kind of Na^+^-channel in stellate cells drives the oscillation of sub-threshold membrane potential in the theta frequency band *in vitro*.^27^ But this intracellular mechanism remains to be validated *in vivo*, where pyramidal cells without such channels exhibit even stronger theta rhythmicity.^13^^,^^28^^,^^29^ Notably, the theta rhythm in the hippocampus can be endogenously generated at a circuit level;^30^ whether a similar network-level mechanism underlies the theta rhythm in mEC and how the oscillation would interact with external rhythmic input remain elusive. Moreover, worthy of attention are the experimental findings that, with pharmacological manipulation of the MS, grid cells lose firing specificity, accompanied by a largely declining theta rhythm in both individual cells and the LFP.^31^^,^^32^ The existing CAN models have failed to fully interpret them, although an external rhythmic input was applied to all neurons in the CAN or other resonance mechanisms were exploited at the single-neuron level.^4^^,^^18^^,^^33^^,^^34^

Here, we explore whether the self-sustained activity and theta oscillation can coexist and vary synergistically in a CAN of grid cells. We built a ring model composed of principal cells (pyramidal and stellate cells) and interneurons, all described by a spiking neural model. We showed how periodic bump attractors and the theta rhythm coexist given the specific synaptic connectivity and revealed the respective contributions of excitatory and inhibitory synaptic currents. We compared the stability of bump attractors and theta rhythm under diverse conditions, including recurrent excitation among principal cells, AMPA receptor-mediated synaptic currents, and structured synaptic connectivity between interneurons. The impact of the MS input to interneurons on network behavior was probed in detail. Multiple experimental observations were accounted for, while testable predictions were also made.

## Results

### Sustained firing activity in the continuous attractor network model

**Figure 1:**
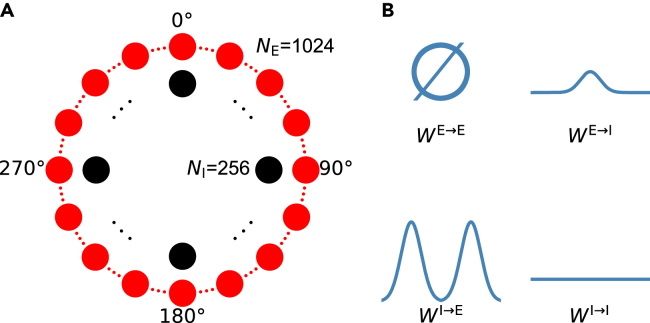
Schematics of the ring model (A) Network architecture. Principal cells (red circle) and interneurons (black circle) are uniformly located on a separate ring and labeled by their angular positions. (B) Profiles of synaptic connectivity within the network. Unless otherwise specified, there is no direct connection between principal cells, and interneurons are uniformly connected. The strength of connectivity between a principal cell (E) and an interneuron (I) depends on their angular difference.

The superficial layers of the mEC contain large numbers of excitatory principal cells and inhibitory interneurons. In previous 2D CAN models, cells are uniformly distributed on a sheet and recurrently connected,^16^^,^^35^ while their firing activities are approximated by gating variables of specific synaptic receptors such as NMDA receptors (NMDARs). As limited mutual excitation between principal cells was found experimentally,^35^^,^^36^ there is usually no direct connectivity between excitatory neurons in these models, and the network is dominated by recurrent inhibition. As neurons on opposite sides of the sheet are interconnected as neighbors, this sheet forms a torus in topology, and the synaptic strength between neurons relies on their distance on the torus. Given strong excitatory input, a hexagonal lattice of bell-shaped activation bumps can be elicited and is able to shift as a whole when the animal runs in the 2D space. Accordingly, individual cells display a hexagonal lattice of firing fields. It is the specific synaptic connectivity profiles, inhibition-dominated recurrent interaction, and strong external input that are essential to the CAN model of grid cells. As the current study aims to elucidate the roles for neurons at different angular positions in maintaining persistent and rhythmic firing, a 1D ring model incorporating the above essential features serves as a good starting point (Figure 1A; STAR Methods). We do not anticipate that a 2D version of our model would act in a qualitatively different manner. Indeed, similar simplifications have been made to explore the dynamics of synapses and membrane potentials of grid cells, and the conclusions with 1D models are similar to those with 2D models.^34^^,^^37^

**Figure 2:**
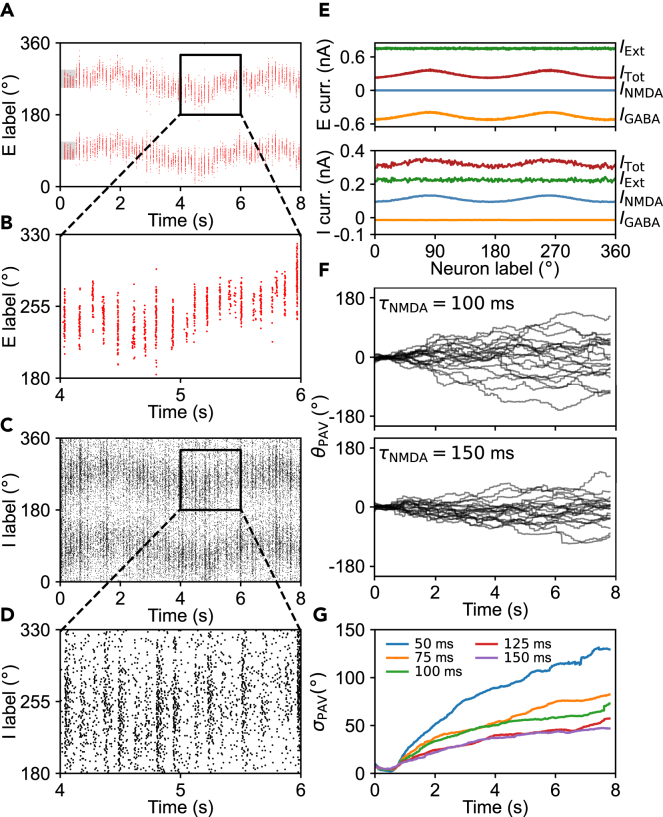
Sustained firing activity in the network (A) One example of raster plot for principal cells. 1,024 principal cells are labeled by their angular positions and arranged along y axis in order. Red dots on each row mark spikes of that neuron. The gray shadows indicate where and when a brief current is applied. Two bump attractors are 180° apart and roughly identical in firing and drifting. (B) Enlarged view of the black rectangle in (A). The cells within each attractor fire synchronously. (C) Raster plot for interneurons from the same simulation as in (A). 256 interneurons are labeled by their angular positions and arranged along y axis in order. Black dots on each row indicate spikes of that neuron. (D) Enlarged view of the black rectangle in (C). (E) Sources of current input to principal cells (upper) and interneurons (lower) labeled by their angular positions; each input is an average over the period of 1.5–2.5 s from the same simulation as in (A). For each cell, *I*_NMDA_, *I*_GABA_, *I*_Ext_, *I*_Tot_ refer to the NMDAR-mediated current, GABAR-mediated current, external current, and total current, respectively. (F) Time courses of the angle of the population activity vector (*θ*_PAV_) on 20 trials for different *τ*_NMDA_. (G) Temporal evolution of the standard deviation of *θ*_PAV_ (*σ*_PAV_) for distinct *τ*_NMDA_. 100 trials were simulated in each case. See also Figure S1.

Here, each unit is described by the leaky integrate-and-fire (LIF) model, and neurons are connected in a manner analogous to that in the CAN model of gating variables^35^^,^^37^ (Figure 1B; STAR Methods). In the default simulation protocol, each cell is subjected to a constant excitatory background input $I_{\text{Back}}^{E}$ ($I_{\text{Back}}^{I}$) plus independent noise, and each interneuron also receives a constant inhibitory current from the MS (*I*_MS,0_). Two bump attractors can spontaneously arise and persist in the network without mutual excitation between principal cells; to initialize the bump attractors centered around 90° and 270° across trials, an additional brief current of 200 pA is applied to some principal cells for 500 ms (Figure 2A). Activated principal cells have a maximal mean firing rate of 3.6 Hz over the period of 1–8 s. The two attractors are separated by 180° and roughly identical in activity; they can be regarded as a minimal periodic structure on the ring, analogous to a hexagonal bump lattice on the torus in the 2D network.^37^ The cells within each attractor discharge synchronously to various extents (Figure 2B). Owing to the structured connectivity from principal cells to interneurons, the interneurons with similar angular locations to the principal cells firing persistently display elevated firing (Figure 2C), with higher firing rates and weaker synchronization than those principal cells (Figure 2D). Together, the network is endowed with sustained activity as in 2D networks.

This persistent activity is evoked and maintained due to the strong external input and slowly decaying NMDAR-mediated currents from principal cells to interneurons (Figure 2E). When a cluster of principal cells are first activated, they excite the interneurons at similar angular positions, and the excitation persists for a relatively long time period. At $\mu_{I\to E}$ = 90° (half the angular distance between the two maxima in the profile of $W^{I\to E}$; see Equation 10), these activated interneurons strongly suppress the principal cells 90° apart, which stabilizes the firings of the previously activated principal cells, giving rise to bump attractors. As a result of overall competition among principal cells, two clusters separated by 180° fire persistently, and the others fire sporadically. The number of concurrent bump attractors is mainly governed by $\mu_{I\to E}$; more attractors can be elicited by decreasing $\mu_{I\to E}$ and adjusting other parameters (Figures S1A–S1C).

On the other hand, the stability of bump attractors is affected by the characteristics of NMDAR-mediated current, e.g., its relaxation time *τ*_NMDA_ (Equation 6). Owing to the stochasticity of external input, the angle *θ*_PAV_ of the population activity vector (PAV), which is the Rayleigh vector of angular positions of all principal cells weighted by their firing rates (see STAR Methods), can drift randomly over time, but the drift is markedly repressed for sufficiently large *τ*_NMDA_ (Figure 2F). The magnitude of the PAV fluctuates slightly around the mean: its coefficient of variation over 1–8 s is 0.142 ± 0.022 and 0.146 ± 0.012 (mean ± S.D., here and thereafter) for *τ*_NMDA_ = 100 ms and 150 ms, respectively. The standard deviation of *θ*_PAV_ tends to rise over time, but the whole trace downregulates with increasing *τ*_NMDA_ in the range of 50–150 ms (Figure 2G). Overall, the structured synaptic connectivity and slow NMDAR-mediated currents underlie stable attractor dynamics.

### Theta rhythm in neural firing

**Figure 3:**
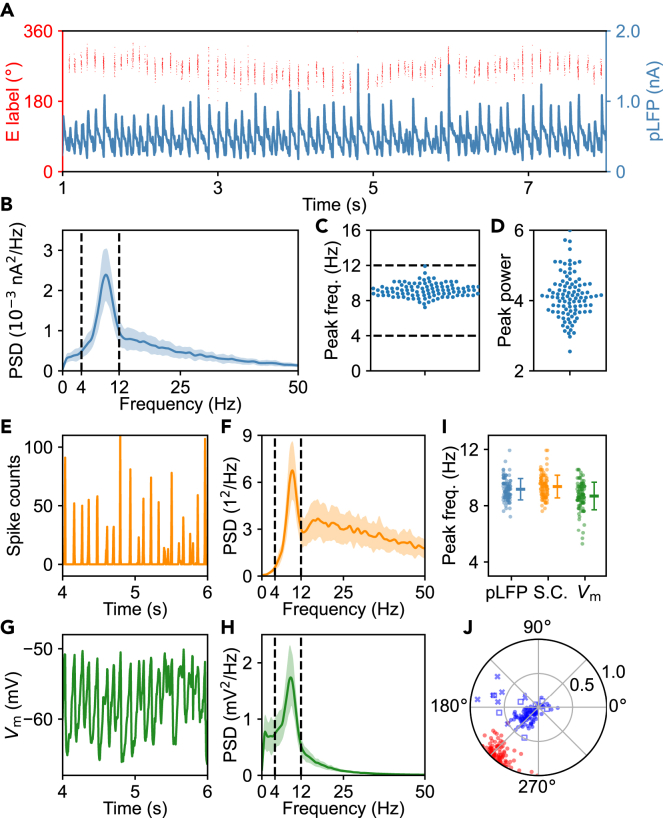
Theta rhythm in the activity of principal cells (The data are from the same simulations as in Figure 2) (A) Time series of spike trains within a bump attractor (red dots) and the LFP proxy (pLFP; blue line). The pLFP is defined as the mean of *τ*_GABA_-delayed GABAR-mediated synaptic currents over all principal cells. (B) PSD of the pLFP averaged over 100 trials. The light shadow marks the standard deviation; the region between two dashed lines indicates the theta frequency range (here and thereafter). (C) Swarm plot of the primary peak frequency in the PSD of pLFP on 100 trials (9.17 ± 0.76 Hz). (D) Swarm plot of the primary peak power in the PSD of pLFP on 100 trials (4.12 ± 0.62). (E) Time course of the spike counts from all principal cells in bins of 5 ms. (F) Averaged PSD of the spike counts from all principal cells in bins of 5 ms over 100 trials. (G) Membrane potential of the principal cell at 90° during a 2-s period. (H) Averaged PSD of the membrane potential of the principal cell at 90° over 100 trials. (I) Strip plots summarizing the peak frequency for three quantities: the pLFP (9.17 ± 0.76 Hz), spike counts from all principal cells in bins of 5 ms (S. C.; 9.36 ± 0.81 Hz), and membrane potential of the 90° principal cell (V_m_; 8.69 ± 0.98 Hz), across the same 100 trials. The bars at the right indicate the mean and standard deviation. (J) Phase-locking index ***Φ*** of the 90° principal cell (red circle) firing with respect to the pLFP on 100 trials. Blue dots, crosses and squares denote the experimental data in (Lepperød et al.,^39^), (Ray et al.,^13^) and (Hafting et al.,^2^), respectively. See also Figures S1–S4.

To verify the rhythm in neural activity, we performed power spectral density (PSD) analyses of multiple time-series signals (see STAR Methods). We first calculated the mean of *τ*_GABA_-delayed GABAR-mediated synaptic currents over all principal cells (Equation 18). This quantity can be taken as a proxy of the LFP (pLFP), since it was shown that this synaptic current in the LIF network closely matches the LFP signal produced in a morphologically realistic 3D network model.^38^ The pLFP obviously exhibits a rhythm (Figure 3A), and the PSD of its temporal evolution over 1–8 s is calculated and averaged over 100 independent trials, showing a characteristic peak around 9.18 Hz (Figure 3B). The primary peak frequency on individual trials always falls within the theta range (9.17 ± 0.76 Hz) (Figure 3C), while the primary peak power, defined as the power in a 2-Hz bin centered at the primary peak frequency divided by that in the range of 0–50 Hz, is 4.12 ± 0.62 (Figure 3D). Notably, this theta rhythm is coherent across a long time period (Figure S2).

Owing to the limited simulation time, individual neurons may discharge a small number of spikes, resulting in a rather sparse auto-correlogram (Figure S3A) and making it hard to determine the rhythm in their spike trains. Thus, we counted the spikes from all principal cells in time bins of 5 ms (Figure 3E) and took this series for PSD analysis (Figure 3F); the primary peak frequency is 9.36 ± 0.81 Hz. Moreover, the sharply valued spike count indicates that activated principal cells are strongly synchronized. We also took the membrane potential of the principal cell at 90° (Figure 3G) for PSD analysis (Figure 3H), and the primary peak frequency is 8.69 ± 0.98 Hz. Other principal cells, including those beyond the attractors, exhibit the similar rhythm in membrane potential (Figures S3B and S3C). Similarly, the theta rhythm is manifested in the spike counts from all interneurons in bins of 5 ms and membrane potentials of individual interneurons (Figure S4). Remarkably, the mean theta frequency is similar across different measures (Figures 3I and S4F).

These results validate the theta rhythm in the neural activity. Of note, the firings of neurons within bump attractors are mainly localized near the troughs of the pLFP. This effect is quantified using the Rayleigh vector ***Φ*** of the neural firing phase relative to the pLFP (see STAR Methods). For the principal cell at 90°, its average arg(***Φ***) and |***Φ***| equal 228.9° and 0.883, respectively (Figure 3J); the values of arg(***Φ***) are close to the experimental data,^3^^,^^13^^,^^39^ while |***Φ***| is always greater in simulation. This discrepancy is possibly associated with the limited number of spikes a cell discharges in simulation and the difference between the pLFP and LFP measured experimentally. Interneurons also exhibit phase-locked firing; for the interneuron at 90°, its mean arg(***Φ***) and |***Φ***| separately equal 301.7° and 0.385 (Figure S4G), suggesting a delayed phase and weaker modulation than the corresponding principal cell on average. The theta modulation of interneurons was also reported experimentally.^40^^,^^41^^,^^42^

Similar phenomena are observable in the case of more coexisting attractors where the primary peak frequency tends to be higher (Figures S1D–S1H). Together, the theta rhythm and phase locking can arise endogenously in the network.

### Negative feedback between principal cells and interneurons underlying the theta rhythm

**Figure 4:**
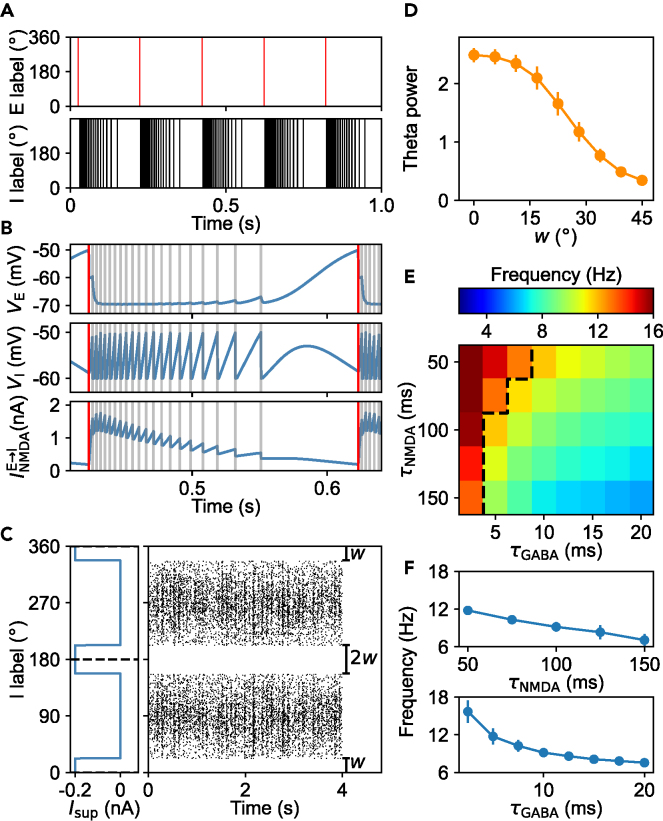
Negative feedback between principal cells and interneurons underlying the theta rhythm (A and B) Neural activity in the deterministic scenario. Here, the constant background input is much less than the default, i.e., $I_{\text{Back}}^{E}$ = 325 pA and $I_{\text{Back}}^{I}$ = 100 pA. (A) Raster plots for principal cells (upper) and interneurons (lower). 1024 principal cells and 256 interneurons are labeled by their angular positions and arranged along y axis in order. Red (black) dots on each row indicate spikes of that principal cell (interneuron). (B) Time courses of membrane potentials of a principal cell (top) and an interneuron (middle) as well as the NMDAR-mediated current to each interneuron (bottom). The red and gray lines indicate spike times of principal cells and interneurons, respectively. (C and D) Neural activity when two clusters of interneurons are suppressed by artificial currents under default parameter settings (i.e., $I_{\text{Back}}^{E}$ = 750 pA and $I_{\text{Back}}^{I}$ = 325 pA, and the noise is included). (C) Raster plot for interneurons (right) together with the profile of suppressive current (left). Some interneurons receive an additional constant current of −200 pA with *w* = 22.5°. (D) Theta power in the PSD of pLFP, averaged over 100 trials, versus *w*. The error bars indicate the standard deviation. (E and F) Dependence of the mean primary peak frequency in the PSD of pLFP on *τ*_NMDA_ and *τ*_GABA_ under default parameter settings. (E) The peak frequency varies with *τ*_NMDA_ and *τ*_GABA._ Parameter sets at the left of the black dashed line engender the frequency higher than 12 Hz. (F) The peak frequency varies with *τ*_NMDA_ at *τ*_GABA_ = 10 ms (upper) or with *τ*_GABA_ at *τ*_NMDA_ = 100 ms (lower). The error bars indicate the standard deviation. See also Figures S5 and S6.

To unveil the mechanism by which the theta rhythm emerges, we ran simulations where initially all neurons have the same membrane potential of −60 mV and receive a constant external input without noise. In this deterministic scenario, all principal cells discharge simultaneously and their firing rate is around 5.07 Hz (Figure 4A). Interneurons also fire synchronously at 101.4 Hz, and their inter-spike intervals vary periodically, with the same rhythm as the firing of principal cells. Thus, the theta rhythm remains despite the absence of attractor dynamics.

A comparison in dynamics of membrane potential between principal cells and interneurons reveals the formation of theta oscillation (Figure 4B). The external input promotes the persistent depolarization of principal cells. The synchronous firing of principal cells delivers a strong NMDAR-mediated current to interneurons, initiating their firing. Each time interneurons discharge, GABAR-mediated inhibitory currents tend to strongly repolarize principal cells, counteracting their depolarization by the external input. With the decay of NMDAR-mediated currents, the total currents are finally too small for interneurons to fire, and then principal cells depolarize to reach the firing threshold and discharge again; a new round of oscillation resumes. Therefore, the theta rhythm results from the negative feedback between principal cells and interneurons.

To further examine the underlying mechanism for theta rhythm in the presence of bump attractors, we suppressed some interneurons by artificially adding inhibitory currents. As interneurons within the attractors are important for attractor maintenance, only interneurons between the two attractors are inhibited. Given the attractors initially centered at 90° and 270°, interneurons with *θ*∈[0°, 0° + *w*]∪[180°*-w*, 180°*+w*]∪[360°*-w*, 360°] receive an additional constant current of −200 pA and are kept from firing (Figure 4C). Compared with the case of *w =* 0*,* this leads to a decrease in inhibitory current to principal cells within bump attractors, and their firing rates increase while the bump attractors become more stable. But the theta rhythm in neural firing weakens as the involved negative feedback between excitatory and inhibitory neurons is reduced. Indeed, with increasing *w*, more interneurons around 0°/360° and 180° are suppressed, resulting in a monotonic decrease in theta power in the PSD of the pLFP, defined as the mean power in 4–12 Hz divided by that in 0–50 Hz (Figure 4D). These results highlight the importance of the negative feedback in mediating theta rhythmicity.

On the other hand, if mutual inhibition between interneurons is markedly strengthened compared with the default settings, the firings of interneurons between the two attractors decrease prominently, leading to enhanced firing of principal cells within the attractors and less drift of the attractors (Figures S5A–S5C). With increasing the inhibitory synaptic conductance, the theta rhythm first declines and then is sequentially replaced by the beta oscillation (12–30 Hz) and gamma oscillation (30–70 Hz) (Figures S5D–S5I). The gamma oscillation in this case is due to strong interactions between interneurons within bump attractors.^43^^,^^44^ Collectively, the theta rhythm is mediated by the interneurons in-between bump attractors.

Experimentally, the typical values of *τ*_NMDA_ and *τ*_GABA_ are separately around 100 ms and 10 ms.^45^^,^^46^ Given the structured connectivity between principal cells and interneurons in the model, the theta rhythm is maintained over a relatively wide range of *τ*_NMDA_ and *τ*_GABA_ (Figures 4E and 4F). Moreover, when the slow NMDAR-mediated currents to interneurons are increasingly replaced with the fast AMPAR-mediated currents (*τ*_AMPA_ = 2 ms), the theta rhythm declines fast and the gamma oscillation gradually arises (Figure S6; see Supplemental materials and methods). Thus, the predominance of NMDARs at recurrent synapses and the long relaxation time of NMDAR-mediated current restrict the oscillation frequency in the theta range.

### Orchestrated variation of the attractor stability and theta rhythmicity

**Figure 5:**
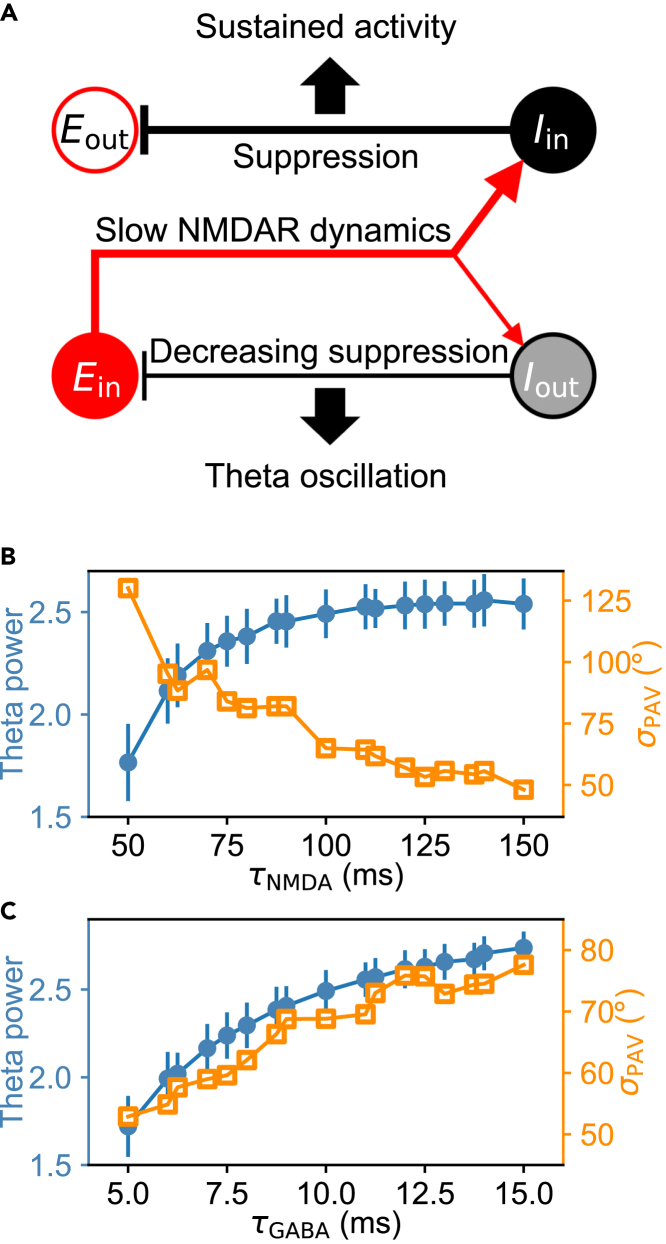
Orchestrated variation of self-sustained activity and theta rhythm (A) Schematic for the formation of sustained activity and theta rhythm. Solid and hollow symbols separately represent neurons within and beyond the bump attractors. Lines with arrows and bars label the activated excitatory and inhibitory synapses, with the line widths indicating their relative strengths. (B and C) Theta power in the PSD of the pLFP (circle) and the standard deviation of *θ*_PAV_ at 7 s (*σ*_PAV_; square) versus *τ*_NMDA_ (at *τ*_GABA_ = 10 ms, B) or *τ*_GABA_ (at *τ*_NMDA_ = 100 ms, C). The average over 100 trials was taken in each case. See also Figures S7–S9.

We have proposed a mechanism for concurrent sustained and rhythmic activities in the network without direct connectivity between principal cells. Under the default parameter settings, principal cells within bump attractors (*E*_in_) deliver strong excitation to interneurons at similar angular positions (*I*_in_), which then primarily inhibit principal cells 90° apart, i.e., beyond the attractors (*E*_out_), but *E*_out_ is unable to influence *E*_in_ via interneurons owing to their low activity, supporting the persistent activity (Figure 5A). Interneurons in between the attractors (*I*_out_) are also excited weakly by *E*_in_ and then inhibit *E*_in_ in return, promoting rhythmic activity. That is, different pools of interneurons serve distinct functions.

Accordingly, the self-sustained firing and theta rhythmicity can vary in an orchestrated manner under diverse conditions. The pLFP theta rhythm is enhanced with increasing *τ*_NMDA_, for example, while the random drift of bump attractors weakens (Figure 5B). The pLFP theta rhythm is also improved with increasing *τ*_GABA_, whereas the random drift of bump attractors enlarges (Figure 5C) mainly owing to the strengthened inhibition from *I*_out_ to *E*_in_. More variation modes are shown in Figure S7 where different parameters are tuned. These orchestrated variations in the persistent and rhythmic activity still originate from the division of labor among interneurons.

When parameter values are adjusted in suitable regimes, the profiles of bump attractors, characterized by their height and full width at half maximum (FWHM) (see STAR Methods), do not vary much in most cases, and the primary peak frequency in the PSD of the pLFP remains in the theta range (Figure S7). Thus, it is appropriate to use only the extent to which bump attractors drift and the theta power to separately characterize the stability of sustained activity and strength of theta rhythmicity.

As there exists limited recurrent excitation among principal cells in superficial layers of the mEC,^36^ we also introduced it to our model. In this case, only NMDAR-mediated currents are considered, and the E-E connectivity strength $W^{E\to E}$ is peaked at zero angular difference (Supplemental materials and methods). With increasing the synaptic conductance, the bump attractors become more stable and the spike times are more concentrated relative to the pLFP rhythm, while the primary peak frequency and theta power in the PSD of pLFP just slightly decrease (Figure S8). Similar changing trends can be observed with increasing $I_{\text{Back}}^{E}$, except that the primary peak frequency rises with $I_{\text{Back}}^{E}$. Overall, the recurrent excitation strengthens the persistent firing but slightly impacts the theta rhythm in our model.

Of note, in the CAN model with structured E-E connectivity and uniform E-I and I-I connectivity, the persistent activity depends on the local excitation and global inhibition.^47^ A stable spontaneous firing state exists along with the bump attractor due to the positive feedback between principal cell,^47^^,^^48^ in contrast to its absence here, while fast oscillation may arise owing to the interplay between the fast AMPAR-mediated recurrent excitation and slower GABAR-mediated inhibition. However, strong rhythmic firing would diminish the persistent activity, because both the self-sustained and oscillatory activity depend on the whole population of interneurons; the self-sustained activity requires the persistent global inhibition from interneurons to stabilize the bump attractor, which is always disturbed by the rhythmic firings of interneurons.^47^ Thus, distinct mechanisms are at play in these two types of networks.

Interneurons are uniformly connected in our default model setup. We further explored two cases of structured I-I connectivity: the connectivity strength $W^{I\to I}$ between two interneurons is either a unimodal or bimodal function of their angular difference (Figure S9 and Supplemental materials and methods). In either case, both the self-sustained activity and theta rhythm are maintained. In the unimodal case, interneurons in the same activated cluster primarily inhibit each other, which may weaken their inhibition of principal cells beyond bump attractors and enlarge the drift of bump attractors. In the bimodal case, interneurons in between the bump attractors are more suppressed by activated interneurons, which tends to reduce their inhibition of principal cells within the bump attractors, leading to more stable attractor dynamics and weaker theta rhythmicity. It is also worth noting that $g_{\text{GABA}}^{I\to I}$, maximal I-I synaptic conductance, is much less than the others, and thus changing the concrete form of $W^{I\to I}$ does not cause large variations. Together, all these results highlight the importance of structured synaptic connectivity between excitatory and inhibitory cells in maintaining the persistent and rhythmic firing.

### Modulation of neural activity by the background input

It has been reported that the MS input exerts a control over neural firing in mEC; blocking the MS input to the mEC largely disrupts both the rhythm and grid patterns of grid cells,^25^^,^^31^^,^^32^ while tuning the MS input evokes distinct firing properties.^18^^,^^39^ The MS primarily delivers GABAergic projection to interneurons.^24^^,^^26^

**Figure 6:**
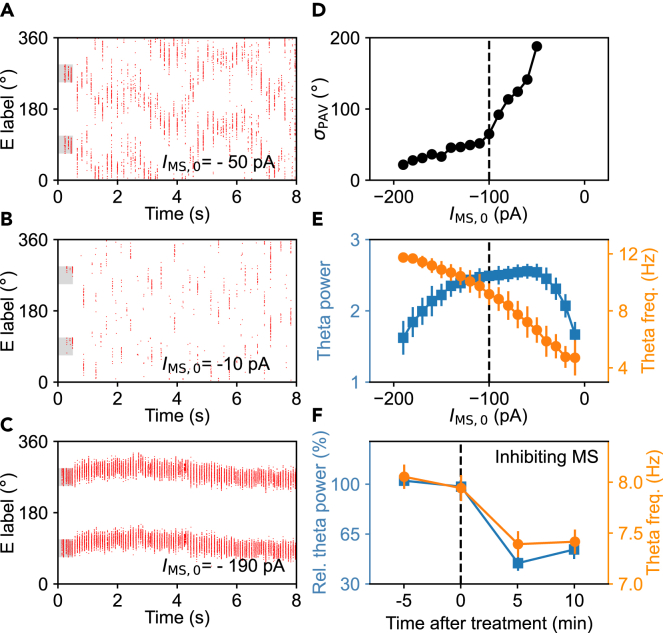
Network behavior under various strengths of MS input (A–C) Raster plots of principal cells for *I*_MS,0_ = −50 pA (A), −10 pA (B), and −190 pA (C). 1024 principal cells are labeled by their angular positions and arranged along y axis in order. Red dots on each row mark spikes of that neuron. The gray shadows indicate when and where the brief current is applied. (D) Standard deviation of *θ*_PAV_ at 7 s (*σ*_PAV_) versus *I*_MS,0_. The dashed line labels the default value of *I*_MS,0_. For |*I*_MS,0_| < 50 pA, there is no attractor distinguishable. (E) Theta power (square) and the theta frequency (peak frequency within the theta range, circle) in the PSD of the pLFP versus *I*_MS,0_. The error bars label the standard deviations. 100 independent trials were simulated in each case. (F) Experimental data from (Koenig et al., 2011^32^). Marks are the same as in (E). See also Figure S10.

We first examined the case where the MS input to each interneuron is a negative constant *I*_MS,0_. The stability of bump attractors weakens with decreasing |*I*_MS,0_| (Figure 6A), and the network fails to maintain persistent firing for |*I*_MS,0_| < 50 pA (Figure 6B). On the contrary, the stability is enhanced with increasing |*I*_MS,0_| (Figure 6C), with the standard deviation of *θ*_PAV_ at 7 s remaining at relatively low levels for |*I*_MS,0_| > 100 pA (Figure 6D). The theta frequency, defined as the peak frequency within the theta range, basically equals the primary peak frequency calculated above in most cases. The theta frequency drops monotonically with decreasing |*I*_MS,0_|, whereas the theta power first rises, varies slightly for 130 > |*I*_MS,0_| > 50 pA, and then declines. With |*I*_MS,0_| deviating from 100 pA, the drop in theta power for |*I*_MS,0_| < 50 pA is mainly caused by the breakdown of the attractors, while that for |*I*_MS,0_| > 130 pA mainly results from that the primary peak frequency on some trials exceeds 12 Hz. Moreover, when the excitatory background and the inhibitory MS input are simulated with synaptic currents from Poisson spike trains,^47^ the network behavior does not change markedly (Figure S10). Together, the strength of inhibitory input from the MS markedly impacts both the persistent firing and rhythmic activity.

These results are generally consistent with the experimental observations: suppressing the input from the MS weakens the theta rhythm (Figure 6F) and breaks the grid pattern.^31^^,^^32^ Indeed, when |*I*_MS,0_| varies from 100 pA (default case) to 10 pA (suppressed case) in our model, both the theta power and theta frequency decrease (Figure 6E), while the bump attractors drift remarkably and finally break down (Figure 6D), resulting in the broken grid pattern. Overall, the deteriorating grid pattern and theta rhythm under suppressed MS input can be accounted for by a decrease in MS input strength.

**Figure 7:**
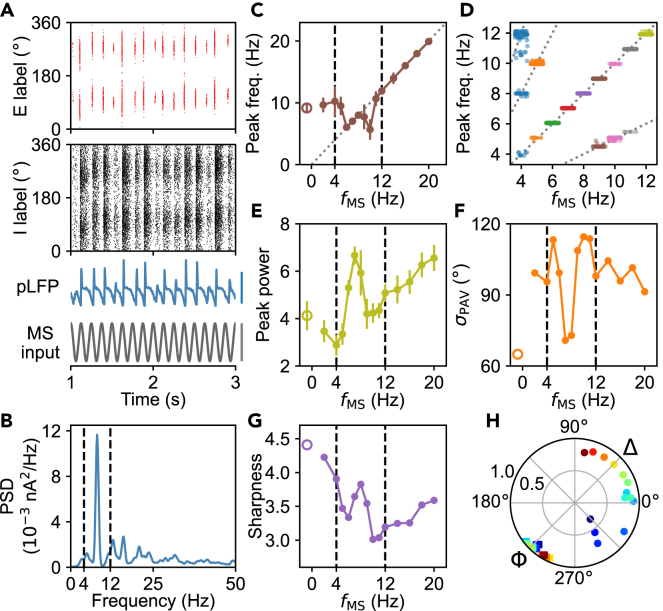
Distinct features of the rhythmic and sustained activities under rhythmic MS input (A) One example of the neural dynamics during 1–3 s at *f*_MS_ = 8 Hz and *A* = 40 pA: raster plots for principal cells and interneurons, the pLFP with the bar at the right indicating 1 nA, and the MS input with the bar at the right indicating 80 pA (from top to bottom). (B) PSD of the pLFP over 1–8 s in the same simulation as in (A). The region between two dashed lines indicates the theta range. (C–G) Features of the rhythmic and sustained activity for different *f*_MS_ at *A* = 40 pA. Shown are the mean primary peak frequency (C) and peak power (E), standard deviation of *θ*_PAV_ (*σ*_PAV_) at 7 s (F), and the sharpness of bump attractors (G). Hollow markers at the left indicate the case of *A* = 0. Results in (C) and (E) are acquired by averaging over 100 trials, with the error bars indicating the standard deviation. Results in (F) and (G) are obtained from the same 100 trials. Strip plot in (D) displays the primary peak frequency in the PSD of pLFP on 100 trials for different *f*_MS_. The slopes of four dotted lines are separately 0.5, 1, 2 and 3 (from bottom to top). (H) Phase-locking indexes of the principal cell at 90° relative to the pLFP theta rhythm (***Φ***, squares) or MS input rhythm (**Δ**, circles) for different *f*_MS_ at *A* = 40 pA. Colors are in order of blue-green-red, corresponding to *f*_MS_ between 2 and 20 Hz in (C–G). In each case, an average over 100 trials was made to obtain the mean. See also Figures S11–S13.

Furthermore, we explored how the rhythmicity of MS input could influence the neural activity. The MS input is assumed to vary periodically— *I*_MS_(*t*) = *I*_MS,0_ + *A*cos(2π*f*_MS_*t*), with *A* and *f*_MS_ denoting the amplitude and frequency of the rhythmic MS input.^18^^,^^33^^,^^34^ At *A* = 0, there exists only one major peak in the PSD of the pLFP on individual trials and the mean primary peak frequency equals *f*_p0_ = 9.17 Hz— intrinsic oscillation frequency (Figure 3). Figures 7A, 7B, S11, and S12 illustrate examples of neural activity for different *A* and *f*_MS_. At *A* = 40 pA, the intrinsic oscillation predominates for *f*_MS_ < 4 Hz, and the mean primary peak frequency *f*_p_ in the PSD of pLFP is around *f*_p0_ (Figures 7C, S11A, and S11B); by contrast, the external input rhythm dominates and *f*_p_ remains around *f*_MS_ for *f*_MS_ > 11 Hz (Figures S11C and S11D). For 4 ≤ *f*_MS_ ≤ 11 Hz, there emerges a resonance effect between the external input and intrinsic oscillation, and peaks in the PSD of pLFP on individual trials may appear around 0.5*f*_MS_ and its integer multiples (Figures S11E–S11H); under strong resonance (6 < *f*_MS_ < 9 Hz), the peak around *f*_MS_ is much higher than others in the PSD (Figures 7D and S11I–S11L). Consequently, the average of primary peak frequency over 100 trials exhibits a complicated dependence on *f*_MS_ (Figure 7C). Similarly, the peak power has a local maximum around *f*_MS_ = 7 Hz because of the strong resonance effect (Figure 7E). By contrast, at *A* = 100 pA the network displays a different behavior, always acting as a forced oscillator; peaks always appear at multiple integers of *f*_MS_ or 0.5*f*_MS_ in the PSD of pLFP on individual trials, and the major peak is located around *f*_MS_ (Figure S12).

At *A* = 40 pA, the bump attractors have minimal random drifts and maximal sharpness when *f*_MS_ is between 7 and 8 Hz, which may be called resonance frequency evoking the maximal resonance effect (Figures 7F and 7G); owing to the involved complex interactions, this frequency may be different from *f*_p0_. For a much larger amplitude of the MS input, such as *A* = 100 pA, bump attractors cannot persist for most *f*_MS_ except for *f*_MS_ around the corresponding resonance frequency (Figures S12C, S13C, and S13D). Overall, the external rhythm disfavors the robustness of bump attractors, but the resonance effect can weaken the deterioration.

Although the phase precession was suggested to arise from the interference between an external and an endogenous theta oscillation with slightly different frequencies,^5^^,^^11^^,^^19^ it is absent in our model. Instead, here the phase locking is rather robust; irrespective of *f*_MS_, spikes of both principal cells and interneurons within bump attractors are always locked to the pLFP theta rhythm although the theta rhythm is not dominant in pLFP in some cases (Figures 7H and S13E–S13H). Moreover, when the rhythmicity of MS input is not too weak (otherwise, it hardly influences the network dynamics), spikes of both principal cells and interneurons are also locked to the input rhythm but with a varying locked phase (Figures 7H and S13I–S13L). Thus, activated principal cells and interneurons are phase-locked to both the pLFP theta rhythm and MS input rhythm.

Notably, part of the results above agrees with the experimental observation.^39^ Experimentally, the parvalbumin-positive (PV^+^) interneurons in MS are stimulated at the frequency of 11 Hz or 30 Hz; the resulting peak frequency and peak power in the PSD of mEC LFP and the spiking of neurons locked to the input rhythm can be accounted for by our results for *f*_MS_ ≥ 11 Hz (Figures 7C, 7E, and 7H). Changes in neural behavior within other *f*_MS_ ranges remain to be experimentally validated.

## Discussion

In this study, we investigated the intrinsic theta rhythm in the CAN model with a grid-cell-like connectivity profile. Without recurrent excitation among principal cells, the connectivity between principal cells and interneurons has to be structured for generating bump attractors, which necessitates the labor division among interneurons. Interneurons within attractors receive strong excitation from principal cells within the attractors and then inhibit principal cells beyond. Given the slow dynamics of NMDAR-mediated currents, such excitation and the resulting inhibition can persist, stabilizing the bump attractors. Interneurons beyond attractors also receive sufficient excitation from principal cells within attractors and then inhibit them in return, and the resulting negative feedback allows for the theta oscillation. Together, both the structured synaptic connectivity and slow NMDAR dynamics are indispensable for the CAN model with theta rhythm.

Our work reproduces several experimental observations. First, the theta rhythms in the pLFP and spike trains of activated principal cells are nearly anti-phased, similar to the experimental reports that the spikes of grid cells are locked near troughs of the LFP.^3^^,^^13^^,^^39^ Second, the declining theta rhythm and broken bump attractors due to reduced MS input agree with the experimental results when inputs from the MS are blocked.^25^^,^^31^^,^^32^ Finally, given the rhythmicity of MS input at the upper end of or far outside the theta range, neural firings are locked to the MS input while bump attractors are maintained.^39^ All these results support a network-level mechanism for theta rhythm in a local circuit of the mEC.

The periodic bump attractors and rhythmic activity are linked inherently in our model, different from previous studies where the theta rhythm was induced externally.^18^^,^^33^^,^^34^^,^^49^ Here, the theta rhythm is endogenous and coexists stably with bump attractors, independent of specific pacemakers or rhythmic inputs.^26^ The current theoretical framework could be exploited to further probe the spatiotemporal firing patterns of grid cells and their role in spatial navigation. The conclusions drawn here may be enlightening for exploring cognitive processes involving both self-sustained and rhythmic activity, such as working memory.^50^^,^^51^

Two testable predictions can be made here. First, we underscore the roles for interneurons and NMDARs in generating the theta rhythm besides grid patterns.^35^^,^^52^ Thus, suppression of interneurons or blockade of NMDARs in the mEC probably leads to breakdown of both the grid pattern and theta rhythm. Part of the prediction has been validated: the stability of grid pattern is reduced after the ablation of NMDARs in the retro-hippocampus region, and the LFP theta rhythm significantly weakens when NMDARs in mEC are blocked.^53^^,^^54^ Notably, the theta rhythm still retains in these two experiments, probably resulting from the MS input. Second, there exists a frequency around the intrinsic theta frequency, with which stimulating the interneurons in mEC may elicit strong theta oscillation in the LFP (e.g., the peak power in its PSD takes a local maximum) and stable bump attractors with a locally minimal drift. Experimentally, such modulation of rhythmic input might be realized by tuning the MS PV^+^ cells via optogenetic methods.^39^ The validation of these predictions would advance the understanding of grid cells.

Two assumptions are critical for the presented results: the NMDAR-mediated current and synaptic connectivity profiles. NMDARs have proved a dominating receptor mediating excitatory synaptic currents in layer II of the mEC^45^ and underlying persistent activity in attractor networks.^47^^,^^55^ Different from $W^{E\to I}$, $W^{I\to E}$, and $W^{I\to I}$ adopted here, other connectivity profiles also contribute to grid patterns.^18^^,^^33^^,^^34^ The main difference lies in the angular bias in E→I and I→E connectivity profiles. This will only impact which pools of interneurons are excited to suppress principal cells beyond the bump attractors, and thus division of interneurons via structured synaptic connectivity can still be realized in these models to support neural oscillation. Overall, our assumptions have a solid experimental and theoretical basis.

Although the MS is involved in modulation of phase precession in mEC,^56^ our results suggest that it may not directly arise from the interplay between the intrinsic rhythm and external input rhythm; we speculate that additional inputs related to spatial information might play a role. Possibly, real-time inputs from speed cells may bring about a shift in bump attractors over time, underpinning the phase precession.^57^ The deviated ramps in the membrane potential of single neurons when a rat runs through one firing field might reflect a current tuning.^4^^,^^20^^,^^58^ Theoretical works also suggested that the phase precession was related to movements of bump attractors.^34^^,^^59^

Note that other kinds of oscillation exist in mEC and interact with the theta rhythm, such as the theta-nested gamma oscillation.^18^ Although gamma oscillation arises in our model due to fast AMPAR-mediated currents^55^ or GABAR-mediated currents,^43^^,^^44^ its relationship with the theta rhythm was not explored in detail here. Different types of interneurons have distinct properties of firing and projection, affecting the spatiotemporal features of grid cells in different manners.^60^^,^^61^ It is worthy to further explore the functions of diverse interneuron types. The random drift of bump attractors over time is inevitable in CAN models. It could be suppressed by sensory inputs such as those from environmental boundaries.^62^ Our results suggest that it can be repressed by tuning various parameters, such as the time constant of NMDARs and applying a rhythmic input with specific frequency to interneurons. It would be interesting to systematically explore how bump attractors could be stabilized. These issues may prompt further work for more advanced models.

To conclude, in the CAN model with structured connectivity between principal cells and interneurons, the theta rhythm can naturally arise along with periodic bump attractors, and their variations are orchestrated under various conditions. A remarkable phase locking emerges between the pLFP and spike trains of both principal cells and interneurons within bump attractors. The theta rhythm gets stronger and the bump attractors become more stable when the frequency of the MS input is near the intrinsic frequency. This study sheds fresh light on the association of attractor and rhythm dynamics in grid cells.

### Limitation of the study

The current model is a 1D network of grid cells. To fully interpret the spatiotemporal firing pattern of grid cells, it is still necessary to construct a 2D network model.

## STAR★Methods

### Key resources table

**Table:** 

REAGENT or RESOURCE	SOURCE	IDENTIFIER
**Software and algorithms**		

Python	https://www.python.org/	RRID:SCR_008394
Numpy	https://numpy.org/	RRID: SCR_008633
Scipy	https://www.scipy.org/	RRID: SCR_008058
Matplotlib	https://matplotlib.org/	RRID: SCR_008624
Original code	This paper	https://github.com/NJUTBP/Intrinsic_Theta_Oscillation_Grid_Cell_Network

### Resource availability

#### Lead contact

Further information and requests for resources should be directed to and will be fulfilled by the Lead Contact, Feng Liu (fliu@nju.edu.cn).

#### Materials availability

This study did not generate new unique reagents.

### Method details

#### Model setup

The network consists of *N*_E_ = 1024 excitatory principal cells (E) and *N*_I_ = 256 inhibitory interneurons (I). They are uniformly distributed on a separate ring, and each neuron is labeled by its angular position *θ*. All neurons are described by the LIF model:

(Equation 1) $$C_{m}\frac{dV_{m}\left(t\right)}{dt}=-g_{L}\left(V_{m}\left(t\right)-V_{L}\right)-I_{syn}\left(t\right)+I_{ext}\left(t\right)\text{,}$$

where *V*_m_, *C*_m_, *g*_L_ and *V*_L_ are the membrane potential, membrane capacitance, leaky conductance and resting potential, respectively. *I*_syn_(*t*) is the total recurrent synaptic current. *I*_ext_(t) is the external current, comprising excitatory and inhibitory inputs from outside the network and the noise. When *V*_m_ reaches the threshold *V*_th_, a spike is fired and *V*_m_ is reset to *V*_reset_ for a refractory period *τ*_ref_. For principal cells, *C*_m_ = 0.5 nF, *g*_L_ = 25 nS, *V*_L_ = -70 mV, *V*_th_ = -50 mV, *V*_reset_ = -60 mV, and *τ*_ref_ = 2 ms; for interneurons, *C*_m_ = 0.2 nF, *g*_L_ = 20 nS, *V*_L_ = -70 mV, *V*_th_ = -50 mV, *V*_reset_ = -60 mV, and *τ*_ref_ = 1 ms.^17^^,^^48^

#### Synaptic currents

Interneurons receive excitatory postsynaptic currents (EPSCs) and inhibitory postsynaptic currents (IPSCs) from principal cells and interneurons, respectively. Owing to limited recurrent excitation,^35^^,^^36^^,^^52^^,^^63^ principal cells receive only IPSCs from interneurons in most simulations. In default setup of the model, the total synaptic current to neuron *i* reads:

(Equation 2) $$I_{syn,i}^{E}\left(t\right)=I_{i}^{I\to E}\left(t\right)\text{,}$$

(Equation 3) $$I_{syn,i}^{I}\left(t\right)=I_{i}^{E\to I}\left(t\right)+I_{i}^{I\to I}\left(t\right)\text{.}$$

IPSCs are mediated by GABA_A_ receptors (GABARs), while EPSCs can be mediated by AMPA receptors (AMPARs) and NMDA receptors (NMDARs).^45^^,^^64^^,^^65^ Considering that only NMDAR-mediated currents are indispensable for persistent firing activity^55^ and the primary role for NMDARs in the superficial mEC layer,^45^ only NMDAR-mediated currents are included here for simplicity. The models involving direct excitation between principal cells and AMPAR-mediated currents are presented in Supplemental materials and methods. The synaptic currents are modeled as follows:^66^

(Equation 4) $$I_{i}^{E\to I}\left(t\right)=I_{NMDA,i}^{E\to I}\left(t\right)=\frac{g_{NMDA}^{E\to I}\left(V_{m,i}\left(t\right)-V_{E}\right)}{1+\left[Mg^{2+}\right]\exp\left(-0.062V_{m,i}\left(t\right)\right)/3.57}\sum_{j=1}^{N_{E}}W_{ij}^{E\to I}S_{NMDA,j}\left(t\right),$$

(Equation 5) $$I_{i}^{I\to \alpha }\left(t\right)=I_{GABA,i}^{I\to \alpha }\left(t\right)=g_{GABA}^{I\to \alpha }\left(V_{m,i}\left(t\right)-V_{I}\right)\sum_{j=1}^{N_{I}}W_{ij}^{I\to \alpha }S_{GABA,j}\left(t\right)\text{,}$$

where *α* is *E* or *I*, and *g* is the maximal synaptic conductance (in nS): $g_{\text{NMDA}}^{E\to I}$ = 0.4, $g_{\text{GABA}}^{I\to E}$ = 2.4, and $g_{\text{GABA}}^{I\to I}$ = 0.04. The reversal potentials for excitatory and inhibitory synapses are *V*_E_ = 0 mV and *V*_I_ = -70 mV, respectively, and [Mg^2+^] = 1 mM. The index *j* runs over all presynaptic neurons, *W*_ij_ is the connectivity strength, and *S*_j_ denotes the fraction of channels in the open state.

Given a spike train {*t*_k_}_j_ in the presynaptic neuron *j*, *S*_NMDA,j_ follows slow dynamics:

(Equation 6) $$\frac{dS_{NMDA,j}\left(t\right)}{dt}=-\frac{S_{NMDA,j}\left(t\right)}{\tau_{NMDA}}+\alpha_{NMDA}x_{j}\left(1-S_{NMDA,j}\left(t\right)\right)\text{,}$$

(Equation 7) $$\frac{dx_{j}\left(t\right)}{dt}=-\frac{x_{j}\left(t\right)}{\tau_{x}}+\sum_{k}\delta \left(t-t_{k}\right)\text{,}$$

where *x*_j_(t) is an intermediate gating variable, *τ*_NMDA_ = 100 ms, *α*_NMDA_ = 0.5 kHz,^45^ and *τ*_x_ = 2 ms. *S*_GABA,j_ obeys fast dynamics:

(Equation 8) $$\frac{dS_{GABA,j}\left(t\right)}{dt}=-\frac{S_{GABA,j}\left(t\right)}{\tau_{GABA}}+\sum_{k}\delta \left(t-t_{k}\right)\text{,}$$

with *τ*_GABA_ = 10 ms.^46^

The strength of synaptic connectivity between principal cells and interneurons takes the profile similar to that in previous studies,^18^^,^^37^ depending on their angular difference Δ*θ* = |*θ*_i_ - *θ*_j_|:

(Equation 9) $$W_{ij}^{E\to I}=W^{E\to I}\left(\Delta \theta \right)=J_{-}^{E\to I}+\left(J_{+}^{E\to I}-J_{-}^{E\to I}\right)\exp\left(-\frac{\Delta \theta^{2}}{2\sigma_{E\to I}^{2}}\right)\text{,}$$

(Equation 10) $$W_{ij}^{I\to E}=W^{I\to E}\left(\Delta \theta \right)=G_{I\to E}\;\exp\left(-\frac{{\left(\Delta \theta -\mu_{I\to E}\right)}^{2}}{2\sigma_{I\to E}^{2}}\right)\text{,}$$

with $J_{+}^{E\to I}$ =1.6, $\sigma_{E\to I}$ =30°, $\mu_{I\to E}$ =90°, and $\sigma_{I\to E}$ =30°. $J^{E\to \text{I}}$ and $G_{\text{I}\to E}$ are separately determined by the normalization condition:

(Equation 11) $$\frac{1}{360{}^{\circ}}\int_{0{}^{\circ}}^{360{}^{\circ}}W\left(\Delta \theta \right)d\left(\Delta \theta \right)=1\text{.}$$

For simplicity, the connectivity between interneurons is assumed to be uniform, i.e., $W_{\text{ij}}^{I\to I}=1$. The model involving structured I-I connectivity is described in Supplemental materials and methods.

#### External currents

Both principal cells and interneurons receive excitatory inputs from neighboring areas as background input, such as directly from the MS via glutamatergic and cholinergic projections and indirectly from the hippocampus via deep layers of mEC.^12^^,^^26^ Moreover, interneurons also receive GABAergic input from the MS, which is shown to be rhythmic.^26^ Noise is introduced to mimic both the intrinsic and extrinsic stochasticity.^67^ Thus, the external currents read:

(Equation 12) $$I_{Ext,i}^{E}\left(t\right)=I_{Back}^{E}+I_{Noise,i}\left(t\right)$$

(Equation 13) $$I_{Ext,j}^{I}\left(t\right)=I_{Back}^{I}+I_{MS}\left(t\right)+I_{Noise,j}\left(t\right)\text{.}$$

The excitatory background inputs are constant, i.e., $I_{\text{Back}}^{E}$ = 750 pA and $I_{\text{Back}}^{I}$ = 325 pA. Two cases of MS GABAergic inputs to interneurons are considered: 1) *I*_MS_(t) = *I*_MS,0_ = -100 pA, which is the default setup; 2) *I*_MS_(t) = *I*_MS,0_ + *A*cos(2π*f*_MS_t), where *A* and *f*_MS_ are the amplitude and frequency of the oscillatory input. The noise current is independent of each other and described by the Ornstein-Uhlenbeck process:^67^^,^^68^

(Equation 14) $$\tau_{Noise}\frac{dI_{Noise,i}\left(t\right)}{dt}=-I_{Noise,i}\left(t\right)+\eta_{Noise}\sqrt{\tau_{Noise}}\cdot n_{i}\left(t\right)$$

where *n*_i_(t) is the Gaussian white noise with zero mean and unit standard deviation, *η*_Noise_ = 150 pA (standard deviation of the noise) and *τ*_Noise_ = 2 ms (time constant of the noise). Another form of input and noise currents is also modeled to exclude the dependence of main conclusions on the concrete form of external input (see Supplemental materials and methods).

#### Population activity vector and profile of a bump attractor

A PAV is calculated to track the central location of a bump attractor.^47^ It is defined as the Rayleigh vector of angular positions of all principal cells weighted by their firing rates. There can exist two bump attractors separated by 180° with the default parameters. To track them simultaneously, the angular position of each neuron is reset by doubling (i.e., *θ*_i_´ = 2*θ*_i_), with the range converting from [0°, 360°) to [0°, 720°), which renders the centers of two attractors differ by 360°. Thus, the two attractors overlap when illustrated in a polar coordinate, and the PAV involving all principal cells reads

(Equation 15) $$P=\frac{1}{N_{E}}\sum_{i=1}^{N_{E}}f_{i}\;\exp\left(j\theta_{i}^{\prime }\right)\text{,}$$

where *j* is the imaginary unit and *f*_i_ is the firing rate of neuron *i* over a time bin of 400 ms. The argument of ***P*** denotes the central location of the ‘overlapping’ bump attractors. Thus, the original angular location of either bump attractor, *θ*_n_ (*n* = 1, 2), before doubling is

(Equation 16) $$\theta_{n}=\frac{\arg\left(P\right)+\left(n-1\right)\cdot 360{}^{\circ}}{2}\text{.}$$

For simplicity, *θ*_1_ is always chosen to represent the PVA location, *θ*_PAV_ = *θ*_1_. With the time bin sliding every 5 ms, a series of *θ*_PAV_ can be obtained to illustrate the drift of bump attractors over time.

Three quantities are introduced to characterize the profile of a bump attractor. Its height is defined as the maximal firing rate of neurons within the attractor, its width refers to the FWHM, and its sharpness is defined as follows:^69^

(Equation 17) $$S=\frac{\sum_{i=1}^{N_{E}^{\prime }}F_{i}\cdot {\left(\theta_{i}-\theta_{0}\right)}^{4}/\sum_{i=1}^{N_{E}^{\prime }}F_{i}}{{\left[\sum_{i=1}^{N_{E}^{\prime }}F_{i}\cdot {\left(\theta_{i}-\theta_{0}\right)}^{2}/\sum_{i=1}^{N_{E}^{\prime }}F_{i}\right]}^{2}}\text{,}$$

where $N_{E}^{\prime }=N_{E}/2$, *θ*_i_ and *F*_i_ are the angular position and firing rate of neuron *i*, and *θ*_0_ is the center of the bump attractor. The sharpness can be regarded as an integrated feature of the height and FWHM of the attractor profile to some degree. For bump attractors of a standard Gaussian profile and a plateau profile, the sharpness is 3.0 and 1.8, respectively.

#### Power spectral density analysis

PSD analysis is used to verify the presence of oscillation in network activity and to determine the primary frequency. The analysis is performed with Python using the *signal.welch* function in *scipy* package, involving a hamming window with the window length *L*, number of overlapping samples (*n*_s_), and number of DFT points (*n*_D_). Here, *L* = 256, *n*_s_ = 128, and *n*_D_ = 1024.

To justify the rhythm in neural activity, we took three signals for PSD analysis. First, the mean of $I_{\text{GABA},i}^{I\to E}\left(t-\tau_{\text{GABA}}\right)$ over all principal cells is taken as a proxy of the LFP (pLFP),^38^ i.e.,

(Equation 18) $$pLFP\left(t\right)=\frac{1}{N_{E}}\sum_{i}^{N_{E}}I_{GABA,i}^{I\to E}\left(t-\tau_{GABA}\right)\text{.}$$

The second is the spike counts from all pyramidal cells (or interneurons) in bins of 5 ms. The third is the membrane potential of individual neurons. The sampling rates for the three signals are all 200 Hz.

#### Theta rhythm and modulation

The phase of neural spiking relative to the pLFP is calculated as follows. The pLFP is first zero-phase bandpass filtered, retaining the theta components (4-12 Hz). Hilbert transform is then performed to obtain the instantaneous phase of the filtered wave, with its peak defined as 0° phase (the filtration and Hilbert transform are made using the *signal.sosfiltfilt* and *signal.hilbert* functions in *scipy* package of Python). The phase of the filtered wave at each spike is determined as the theta phase of the spike. A Rayleigh vector of the theta phase evaluates the extent to which the neural firing is locked to a specific pLFP,^13^

(Equation 19) $$\Phi =\frac{1}{N_{s}}\sum_{i=1}^{N_{s}}\exp\left(j\varphi_{i}\right)\text{,}$$

where *j* is the imaginary unit, *N*_s_ is the total number of spikes, and *φ*_i_ labels the theta phase of the *i*th spike. The modulus and argument of ***Φ***, |***Φ***| and arg(***Φ***), characterize the strength and angle of spikes locked to the pLFP, respectively.^28^ |***Φ***| lies between 0 and 1.

Similarly, for rhythmic MS inputs, the phase of the input rhythm at each spike is determined as the input phase of the spike. A Rayleigh vector of the input phase evaluates the extent to which the neural firing is locked to a specific MS input,

(Equation 20) $$\Delta =\frac{1}{N_{S}}\sum_{i=1}^{N_{S}}\exp\left(j\delta_{i}\right)\text{,}$$

where *j* is the imaginary unit, *N*_S_ is the total number of spikes, and *δ*_i_ labels the input phase of the *i*th spike. The modulus and argument of **Δ**, |**Δ**| and arg(**Δ**), characterize the strength and angle of spikes locked to the MS input, respectively. |**Δ**| lies between 0 and 1.

#### Numerical method

A second-order Runge-Kutta method with a time step of 0.02 ms is exploited to numerically integrate ODEs of the model, and the noise current is carefully calculated following Ito integral. The initial membrane potentials of all neurons are randomly valued between -60 and -50 mV, and all gating variables are set to zero unless otherwise specified. Although bump attractors can arise spontaneously in the network without any cue input, a brief current of 200 pA is applied to the principal cells located within [67.5°, 112.5°] and [247.5°, 292.5°] during the first 500 ms of each simulation to control the initial angular position of the bump attractors. Scripts for all simulations and analysis are written with Python. Most parameter values are fixed in simulation; *τ*_NMDA_, *τ*_GABA_, $C_{m}^{E}$, $C_{m}^{I}$, $g_{\text{GABA}}^{I\to E}$, $g_{\text{GABA}}^{I\to I}$, $g_{\text{NMDA}}^{E\to I}$, $J_{+}^{E\to I}$, $\sigma_{E\to I}$, $\sigma_{I\to E}$, *η*_Noise_, *I*_MS,0_, *A*, and *f*_MS_ are altered to explore their influence on network dynamics, while $\mu_{I\to E}$, $\sigma_{E\to I}$, $\sigma_{I\to E}$, $I_{\text{Back}}^{E}$, and $I_{\text{Back}}^{I}$ are adjusted for more bump attractors.

## Data Availability

All data needed to evaluate the conclusions in the paper are present in the paper. Custom python codes for reproducing the simulation and analysis are available at https://github.com/NJUTBP/Intrinsic_Theta_Oscillation_Grid_Cell_Network.
