# Supplementary material for: Intrinsic theta oscillation in the attractor network of grid cells
Source: iScience. 2023 Mar 14;26(4):106351. doi: 10.1016/j.isci.2023.106351 (PMC10050492; doi:10.1016/j.isci.2023.106351)
Supplement: Document S1. Supplemental materials and methods and Figures S1–S13 [file mmc1.pdf]

iScience, Volume *26*

Supplemental information

Intrinsic theta oscillation in the attractor network of grid cells

Ziqun Wang, Tao Wang, Fan Yang, Feng Liu, and Wei Wang

This document contains Supplementary Methods and Supplementary Figures 1-13.

Supplementary Methods

1. Synaptic currents from Poisson spike trains as background and MS input

In this case, $I^{\mathrm{E}}_{\mathrm{Ext}}$ and $I^{\mathrm{I}}_{\mathrm{Ext}}$ in Equations (12) and (13) are replaced by synaptic currents from neurons outside the recurrent network. Specifically, the background input is mediated by AMPARs:

$$I^{\mathrm{Back}\to\alpha}_{\mathrm{AMPA,i}}(t) = g^{\mathrm{Back}\to\alpha}_{\mathrm{AMPA}}(V_{\mathrm{m,i}}(t) - V_{\mathrm{E}})S^{\mathrm{Back}\to\alpha}_{\mathrm{AMPA,i}}, \quad \text{(S1)}$$

with α is E or I, $g^{\mathrm{Back}\to\mathrm{E}}_{\mathrm{AMPA}}$ = 3.9 nS and $g^{\mathrm{Back}\to\mathrm{I}}_{\mathrm{AMPA}}$ = 1.4 nS. Given a presynaptic spike train $\{t_{\mathrm{k,Back}}\}_{\mathrm{i}}$ to neuron i, $S^{\mathrm{Back}\to\alpha}_{\mathrm{AMPA,i}}$ follows fast dynamics:

$$\frac{\mathrm{d}S^{\mathrm{Back}\to\alpha}_{\mathrm{AMPA,i}}}{\mathrm{d}t} = -\frac{S^{\mathrm{Back}\to\alpha}_{\mathrm{AMPA,i}}}{\tau_{\mathrm{AMPA}}} + \sum_k \delta(t - t_{\mathrm{k,Back}}), \quad \text{(S2)}$$

with τ_{AMPA} = 2 ms. The spike trains $\{t_{\mathrm{k,Back}}\}_{\mathrm{i}}$ is taken as a Poisson process with rate F_{Back} = 1.8 kHz, possibly representing 1000 presynaptic neurons firing at 1.8 Hz.

The MS input to interneurons is mediated by GABARs:

$$I^{\mathrm{MS}\to\mathrm{I}}_{\mathrm{GABA,i}}(t) = g^{\mathrm{MS}\to\mathrm{I}}_{\mathrm{GABA}}(V_{\mathrm{m,i}}(t) - V_{\mathrm{I}})S^{\mathrm{MS}\to\mathrm{I}}_{\mathrm{GABA,i}}, \quad \text{(S3)}$$

with $g^{\mathrm{MS}\to\mathrm{I}}_{\mathrm{GABA}}$ = 0.8 nS. Given a presynaptic spike train $\{t_{\mathrm{k,MS}}\}_{\mathrm{i}}$ to neuron i, $S^{\mathrm{MS}\to\mathrm{I}}_{\mathrm{GABA,i}}$ follows the dynamics:

$$\frac{\mathrm{d}S^{\mathrm{MS}\to\mathrm{I}}_{\mathrm{GABA,i}}}{\mathrm{d}t} = -\frac{S^{MS\to\mathrm{I}}_{\mathrm{GABA,i}}}{\tau_{\mathrm{GABA}}} + \sum_k \delta(t - t_{\mathrm{k,MS}}), \quad \text{(S4)}$$

with τ_{GABA} = 10 ms. The spike train $\{t_{\mathrm{k,MS}}\}_{\mathrm{i}}$ is taken as a Poisson process with rate F_{MS} = 0.5 kHz and independent of each other.

2. Recurrent excitations between principal cells

To explore the role for recurrent excitations between principal cells in the network, we added the following $I^{\mathrm{E}\to\mathrm{E}}_{\mathrm{NMDA,j}}(t)$ to Equation (2):

$$I_{\mathrm{NMDA,i}}^{\mathrm{E\to E}}(t)=\frac{g_{\mathrm{NMDA}}^{\mathrm{E\to E}}(V_{\mathrm{m,i}}(t)-V_{\mathrm{E}})}{1+[\mathrm{Mg}^{2+}]\exp\left(-0.062V_{\mathrm{m,i}}(t)\right)/3.57}\sum_{\mathrm{j=1}}^{N_{\mathrm{E}}}W_{\mathrm{ij}}^{\mathrm{E\to E}}s_{\mathrm{NMDA,j}} \tag{S5}$$

The strength of synaptic connectivity also depends on their angular difference $\Delta\theta = |\theta_{\mathrm{i}} - \theta_{\mathrm{j}}|$:

$$W_{\mathrm{ij}}^{\mathrm{E\to E}} = W^{\mathrm{E\to E}}(\Delta\theta) = J_{-}^{\mathrm{E\to E}} + (J_{+}^{\mathrm{E\to E}} - J_{-}^{\mathrm{E\to E}})\exp(-\frac{\Delta\theta^2}{2\sigma_{\mathrm{E\to E}}^2}) \tag{S6}$$

with $\sigma_{\mathrm{E\to E}} = 30°$. $g_{\mathrm{NMDA}}^{\mathrm{E\to E}}$ and $J_{+}^{\mathrm{E\to E}}$ are tunable parameters.

3. AMPA currents at recurrent synapses

To explore the role of AMPARs in the network, we added the following $I_{\mathrm{AMPA,j}}^{\mathrm{E\to I}}(t)$ to Equation (3):

$$I_{\mathrm{AMPA,i}}^{\mathrm{E\to I}}(t) = g_{\mathrm{AMPA}}^{\mathrm{E\to I}}(V_{\mathrm{m,i}}(t)-V_{\mathrm{E}})\sum_{\mathrm{j=1}}^{N_{\mathrm{E}}}W_{\mathrm{ij}}^{\mathrm{E\to I}}s_{\mathrm{AMPA,j}}, \tag{S7}$$

where $S_{\mathrm{AMPA,j}}$ follows the fast dynamics given a spike train $\{t_{\mathrm{k}}\}_{\mathrm{j}}$ in the presynaptic neuron j:

$$\frac{\mathrm{d}s_{\mathrm{AMPA,j}}(t)}{\mathrm{d}t} = -\frac{s_{\mathrm{AMPA,j}}(t)}{\tau_{\mathrm{AMPA}}} + \sum_{\mathrm{k}}\delta(t-t_{\mathrm{k}}). \tag{S8}$$

As in Ref. S1, $g_{\mathrm{AMPA}}^{\mathrm{E\to I}}$ and $g_{\mathrm{NMDA}}^{\mathrm{E\to I}}$ are tuned together to keep unchanged the excitatory charge entering an interneuron at a holding potential of -65 mV. To be specific, for 0%, 20%, 40%, 60%, 80%, and 100% contributions to EPSCs by AMPAR-medicated currents, $g_{\mathrm{AMPA}}^{\mathrm{E\to I}}$ (in nS) is 0.0, 0.158, 0.311, 0.464, 0.616, and 0.769, while the corresponding $g_{\mathrm{NMDA}}^{\mathrm{E\to I}}$ (in nS) is 0.40, 0.32, 0.24, 0.16, 0.08, and 0.0.

4. Structured connectivity between interneurons

To illustrate the influence of structured connectivity between interneurons on network behavior, the connectivity strength is modeled as follows:

$$W_{\mathrm{ij}}^{\mathrm{I}\to\mathrm{I}} = W^{\mathrm{I}\to\mathrm{I}}(\Delta\theta) = G_{\mathrm{I}\to\mathrm{I}}\exp(-\frac{(\Delta\theta - \mu_{\mathrm{I}\to\mathrm{I}})^2}{2\sigma_{\mathrm{I}\to\mathrm{I}}^2}) \quad \text{(S9)}$$

with $\mu_{\mathrm{I}\to\mathrm{I}} = 0°$ or $90°$ and $\sigma_{\mathrm{I}\to\mathrm{I}} = 30°$. $G_{\mathrm{I}\to\mathrm{I}}$ is determined by the normalization condition. At $\mu_{\mathrm{I}\to\mathrm{I}} = 0°$, the connectivity profile is a classic Gaussian profile; at $\mu_{\mathrm{I}\to\mathrm{I}} = 90°$, it is similar to the one acquired via spike-timing dependent plasticity.[S2]

References

S1. Compte, A., Brunel, N., Goldman-Rakic, P.S., and Wang, X.-J. (2000). Synaptic mechanisms and network dynamics underlying spatial working memory in a cortical network model. Cereb. Cortex *10*, 910-923.

S2. Widloski, J., and Fiete, I.R. (2014). A model of grid cell development through spatial exploration and spike time-dependent plasticity. Neuron *83*, 481-495.

Supplementary Figures

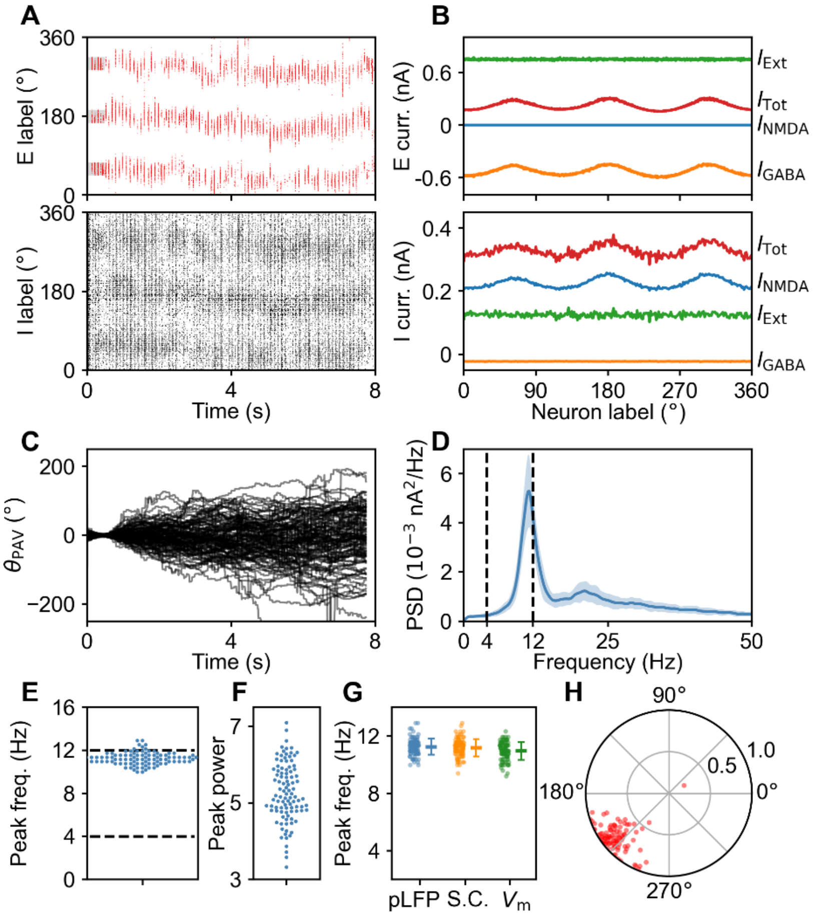

Figure S1. Network behavior in the presence of three bump attractors (related to Figures 2 and 3)

(A) Raster plots for principal cells (upper) and interneurons (lower). 1024 principal cells and 256 interneurons are labeled by their angular positions and arranged along y-axis in order. Red (black) dots on each row mark spikes of that principal cell (interneuron). The gray shadows indicate when and where a brief current is applied. Three bumps are 120° apart and nearly identical in behavior.

(B) Sources of current input to principal cells (upper) and interneurons (lower); each input is an average over the period of 1.5-2.5 s. I_{NMDA}, I_{GABA}, I_{Ext}, I_{Tot} refer to the NMDAR-mediated synaptic current, GABAR-mediated synaptic current, external current, and total current, respectively.

(C) Time courses of the angle of the population activity vector (θ_{PAV}) on 100 trials.

(D) Power spectral density (PSD) of pLFP averaged over 100 trials. The light shadow marks the standard deviation; the region between two dashed lines labels the theta range (here and thereafter).

(E) Swarm plot of the primary peak frequency in the PSD of pLFP on 100 trials.

(F) Swarm plot of the primary peak power in the PSD of pLFP on 100 trials, with the mean and standard deviation equaling 5.27 and 0.757, respectively.

(G) Strip plots summarizing the primary peak frequency for three quantities: the pLFP (11.24 ± 0.57 Hz), spike counts from all principal cells in bins of 5 ms (S. C.; 11.17 ± 0.61 Hz), and membrane potential of the 90° principal cell (V_m; 10.94 ± 0.62 Hz), across the same 100 trials. The bars at the right indicate the mean and standard deviation.

(H) Phase-locking index $\boldsymbol{\Phi}$ of the 90° principal cells (red circle) firing with respect to the pLFP on 100 trials, with its average arg($\boldsymbol{\Phi}$) and |$\boldsymbol{\Phi}$| equaling 219.6° and 0.827, respectively.

The parameter values are the same as those in the case of two attractors except $\mu_{\mathrm{I}\to\mathrm{E}}$ = 60°, $\sigma_{\mathrm{E}\to\mathrm{I}}$ = 16°, $\sigma_{\mathrm{I}\to\mathrm{E}}$ = 16°, $I^{\mathrm{E}}_{\mathrm{Back}}$ = 750 pA, and $I^{\mathrm{I}}_{\mathrm{Back}}$ = 225 pA.

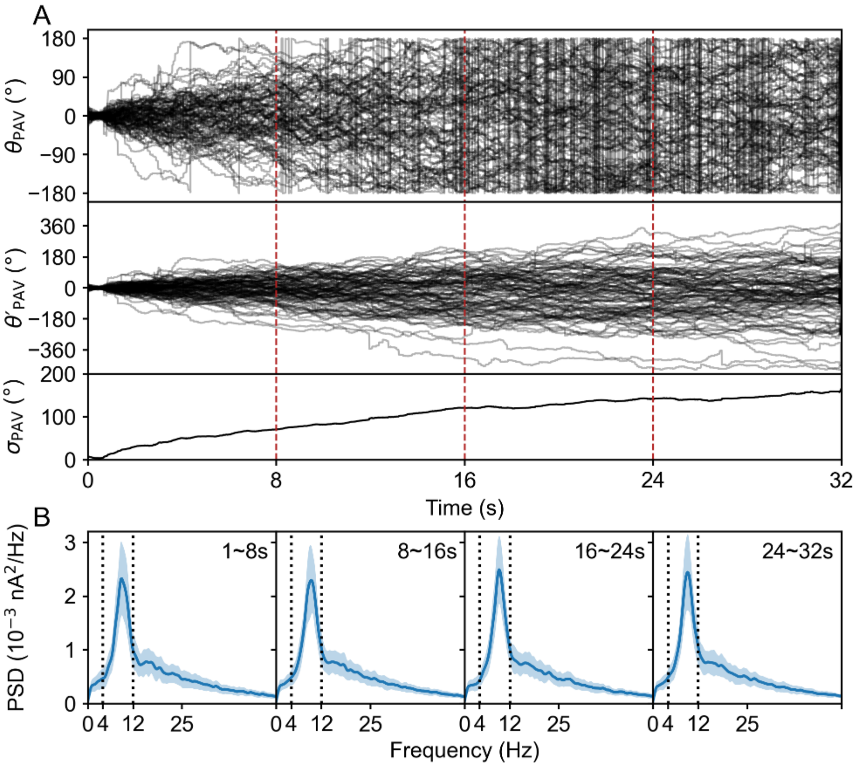

Figure S2. Neural behavior in long-time simulations (related to Figure 3)

(A) Time courses of the angle θ_{PAV} of the population activity vector on 100 trials (upper), the accumulated form of θ_{PAV} (θ'_{PAV}, middle) and the resulting standard deviation σ_{PAV} (lower). Red dashed lines separate the whole simulation into four episodes of 8 s. As θ_{PAV} is restricted between -180° and 180°, its value should subtract (add) 360° once it crosses the upper (lower) bound, whereas θ'_{PAV} is free of such constraint. During long time simulation, θ_{PAV} always crosses the bounds, making it difficult to evaluate its drift over time. Thus, σ_{PAV} is calculated based on θ'_{PAV}.

(B) Power spectral density (PSD) of the pLFP averaged over 100 trials for each episode in (A), with the light shadow labeling the standard deviation. The PSDs are rather consistent, and the primary peak frequency is 9.31 ± 0.89, 9.38 ± 0.88, 9.28 ± 0.78, and 9.21 ± 0.84 Hz sequentially.

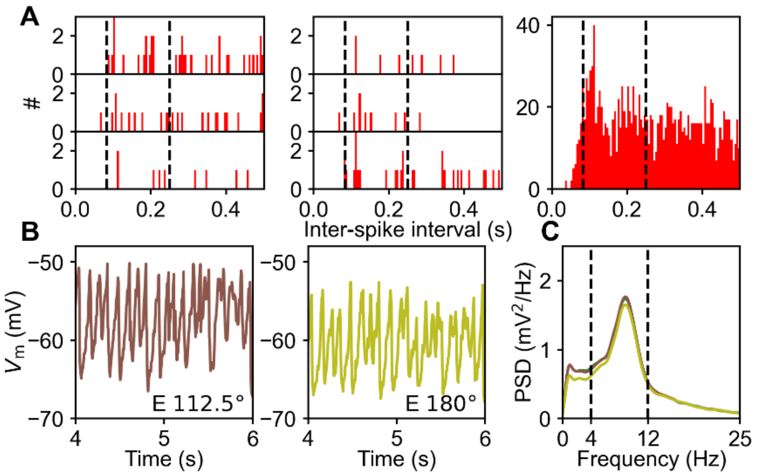

Figure S3. Auto-correlograms and membrane potentials of principal cells (related to Figure 3)

The data here are from the same simulation as in Figure 3.

(A) Spiking auto-correlograms of the principal cell at 90° from 6 different trials (left and middle) and the summation of auto-correlograms from 100 trials (right). The two dashed lines label 1/12 s and 1/4 s.

(B) Membrane potentials of the 112.5° and 180° principal cells during a 2-s period from the same simulation in Figure 3G. They are located at the edge of and outside a bump attractor, respectively.

(C) Power spectrum density (PSD) of the membrane potentials of the two principal cells averaged over 100 trials. The same color code as in B is taken, and the result in Figure 3H is presented in green for comparison.

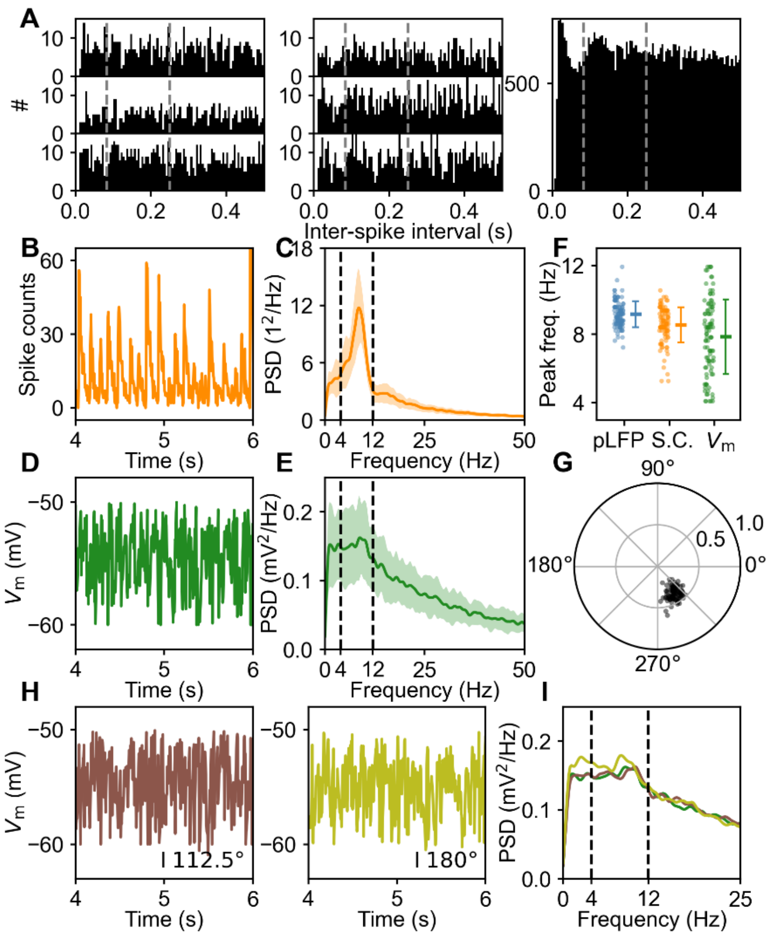

Figure S4. Rhythms in interneurons (related to Figure 3)

The data are from the same simulation as in Figure 3.

(A) Spiking auto-correlograms of the 90° interneuron from 6 different trials (left and middle) and the summation of auto-correlograms from 100 trials (right). The two dashed lines label 1/12 s and 1/4 s.

(B) Spike counts from all interneurons in bins of 5 ms during a 2-s period from the same simulation in Figure 3E.

(C) Power spectrum density (PSD) of the interneuron spike counts averaged over 100 trials, with the light shadow labeling the standard deviation.

(D) Membrane potential of the 90° interneuron during a 2-s period from the same simulation in Figure 3G.

(E) PSD of the membrane potential of the 90° interneuron averaged over 100 trials.

(F) Strip plots summarizing the primary peak frequency for three quantities: the pLFP

(9.17 ± 0.76 Hz), spike counts from all interneurons in bins of 5 ms (S. C.; 8.53 ± 1.02 Hz), and membrane potential of the 90° interneuron (V_m; 7.84 ± 2.18 Hz). The bars at the right indicate the mean and standard deviation.

(G) Phase-locking index $\boldsymbol{\Phi}$ of the 90° interneuron firing with respect to the pLFP over 100 trials, with the mean arg($\boldsymbol{\Phi}$) and |$\boldsymbol{\Phi}$| equaling 301.7° and 0.385, respectively.

(H) Membrane potentials of the 112.5° and 180° interneurons during a 2-s period from the same simulation in (D). They are at the edge of and outside a bump attractor, respectively.

(I) Mean PSD of the membrane potentials of the three interneurons in (D) and (H).

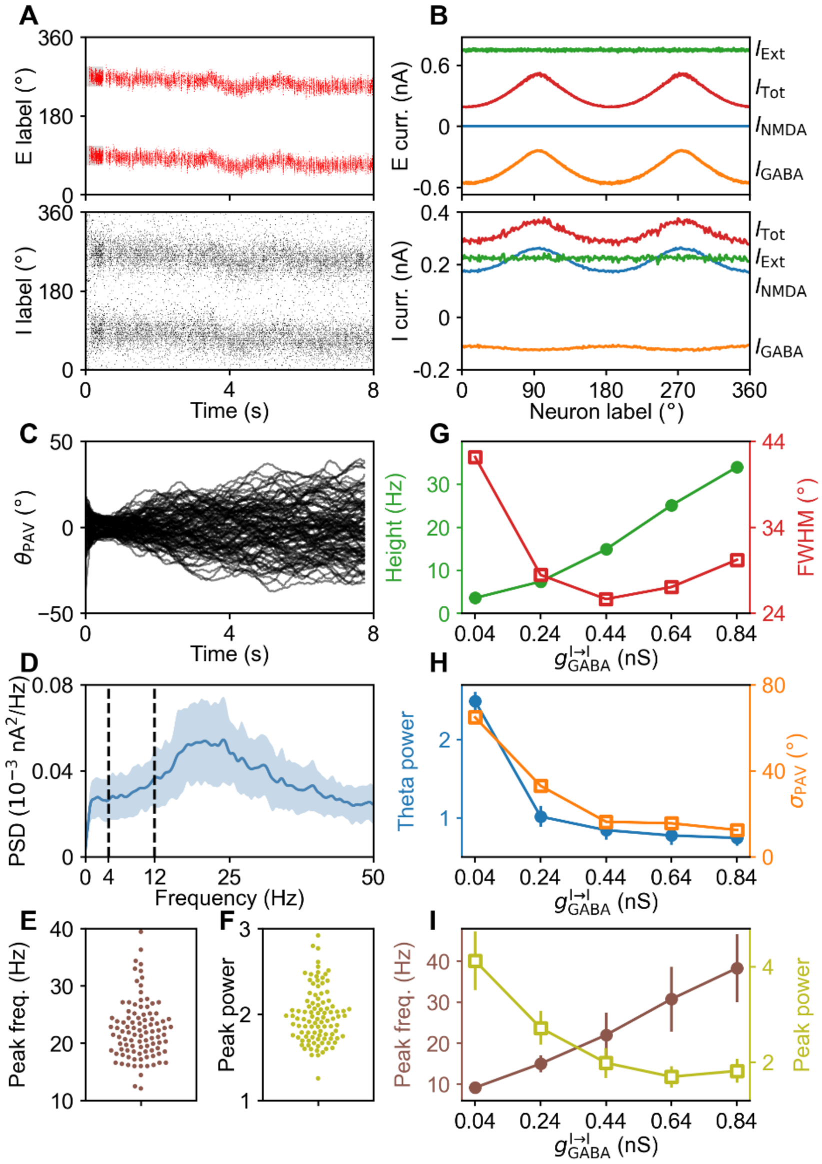

Figure S5. Dependence of network behavior on the inhibitory synaptic conductance $g_{GABA}^{I\to I}$ (related to Figure 4)

(A) Raster plots for principal cells (upper) and interneurons (lower). 1024 principal cells and 256 interneurons are labeled by their angular positions and arranged along y-axis in order. Red (black) dots on each row mark spikes of that principal cell (interneuron). The gray shadows indicate where and when a brief current is applied. $g_{GABA}^{I\to I} = 0.44$ nS in (A-F).

(B) Sources of current input to principal cells (upper) and interneurons (lower).

(C) Time courses of the angle of the population activity vector (θ_{PAV}) on 100 trials.

(D) Power spectral density (PSD) of pLFP averaged over 100 trials. The light shadow marks the standard deviation.

(E) Swarm plot of the primary peak frequency in the PSD of pLFP on 100 trials (22.02 ± 5.42 Hz).

(F) Swarm plot of the primary peak power in the PSD of pLFP on 100 trials (1.99 ± 0.313).

(G) Height (green circle) and full width at half maximum (FWHM, red square) of bump attractors versus $g_{\mathrm{GABA}}^{\mathrm{I \to I}}$.

(H) Theta power (blue circle) in the PSD of pLFP and the standard deviation of θ_{PAV} at 7 s (σ_{PAV}, orange square) versus $g_{\mathrm{GABA}}^{\mathrm{I \to I}}$.

(I) Primary peak frequency (brown circle) and peak power (olive square) in the PSD of pLFP versus $g_{\mathrm{GABA}}^{\mathrm{I \to I}}$.

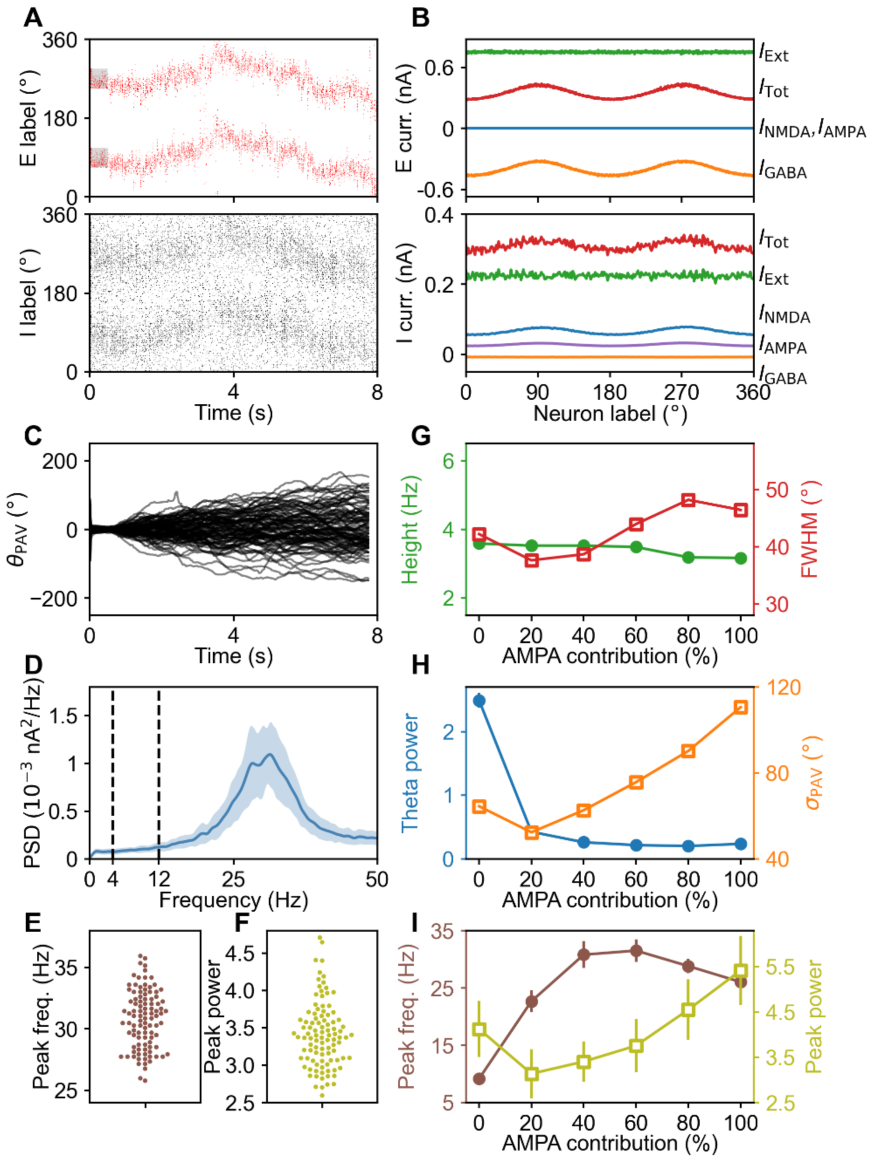

Figure S6. Network behavior with AMPAR-mediated synaptic currents included (related to Figure 4)

(A) Raster plots for principal cells (upper) and interneurons (lower). 1024 principal cells and 256 interneurons are labeled by their angular positions and arranged along y-axis in order. Red (black) dots on each row mark spikes of that principal cell (interneuron). The gray shadows indicate where and when a brief current is applied. In (A-F), a 40% AMPA contribution to EPSCs is held at a holding potential of -65 mV.

(B) Sources of current input to principal cells (upper) and interneurons (lower); each

input is an average over the period of 1.5-2.5 s. I_{NMDA}, I_{AMPA}, I_{GABA}, I_{Ext}, and I_{Tot} refer to the NMDAR-mediated synaptic current, AMPAR-mediated synaptic current, GABAR-mediated synaptic current, external current, and total current, respectively.

(C) Time courses of the angle of the population activity vector (θ_{PAV}) on 100 trials.

(D) Power spectral density (PSD) of pLFP averaged over 100 trials. The light shadow marks the standard deviation.

(E) Swarm plot of the primary peak frequency in the PSD of pLFP on 100 trials (30.80 ± 2.34 Hz).

(F) Swarm plot of the primary peak power in the PSD of pLFP on 100 trials (3.40 ± 0.445).

(G) Height (green circle) and full width at half maximum (FWHM, red square) of bump attractors versus the AMPA contribution to EPSC at a holding potential of -65 mV.

(H) Theta power (blue circle) in the PSD of pLFP and the standard deviation of θ_{PAV} at 7 s (σ_{PAV}, orange square) versus the AMPA contribution to EPSC.

(I) Primary peak frequency (brown circle) and peak power (olive square) in the PSD of pLFP versus the AMPA contribution to EPSC.

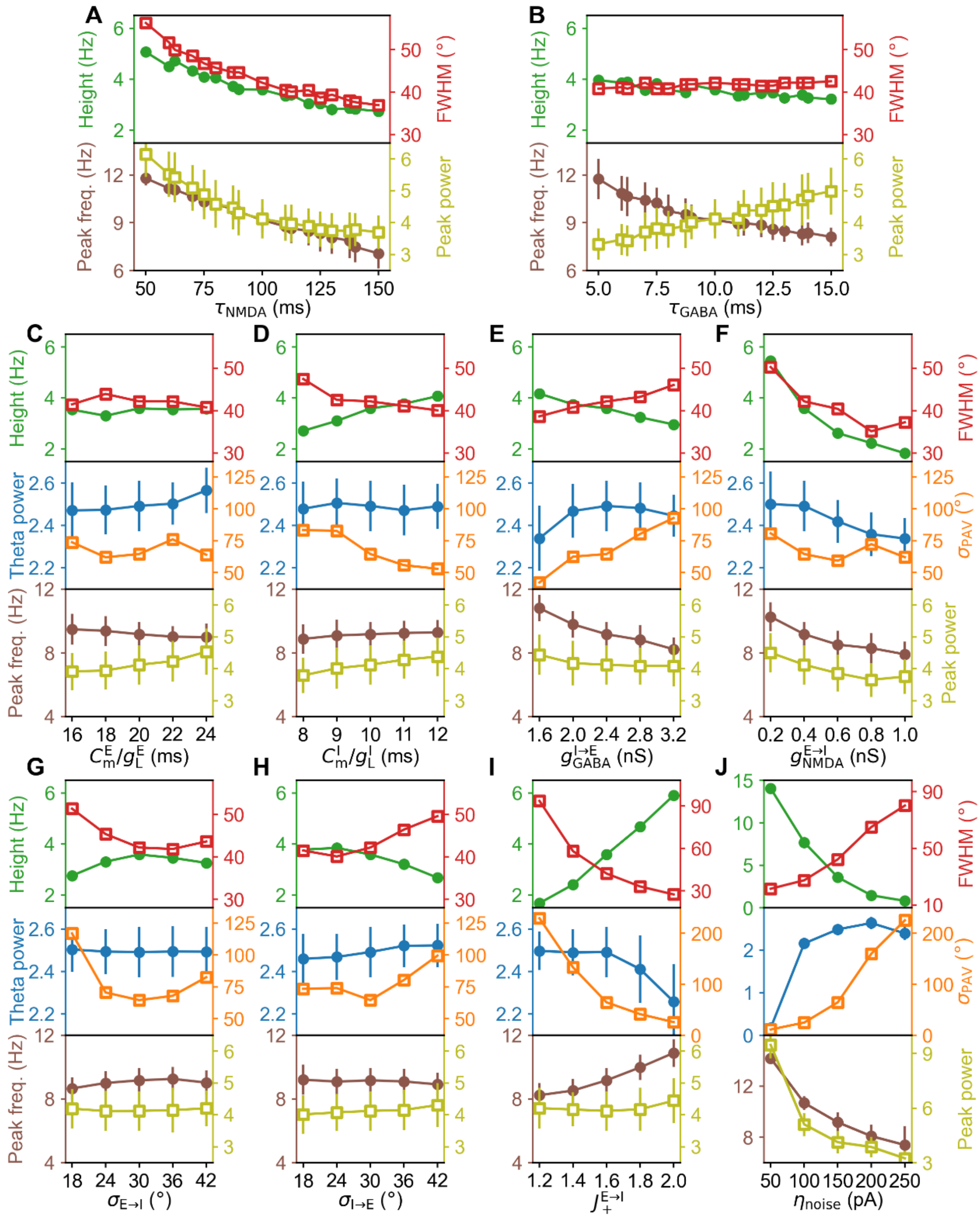

Figure S7. Dependence of sustained and rhythmic activities on various parameters (related to Figure 5)

(A and B) Height (green circle) and full width at half maximum (FWHM, red square) of bump attractors, the primary peak frequency (brown circle) and peak power (olive square) versus τ_{NMDA} (A) and τ_{GABA} (B).

(C-J) Height (green circle, upper) and full width at half maximum (FWHM; red square, upper) of bump attractors, standard deviation of θ_{PAV} at 7 s (σ_{PAV}; orange square， middle), theta power (blue circle, middle), primary peak frequency (brown circle, lower), and peak power (olive square, lower) in the PSD of pLFP versus the time constant of principal cells (C_m^E / g_L^E ; C), that of interneurons (C_m^I / g_L^I ; D), $g_{GABA}^{I\rightarrow E}$ (E), $g_{NMDA}^{E\rightarrow I}$ (F), $\sigma_{E\rightarrow I}$ (G), $\sigma_{I\rightarrow E}$ (H), $J_+^{E\rightarrow I}$ (I), and η_{Noise} (J). In each case, only one parameter is varied while the others are kept at the default values.

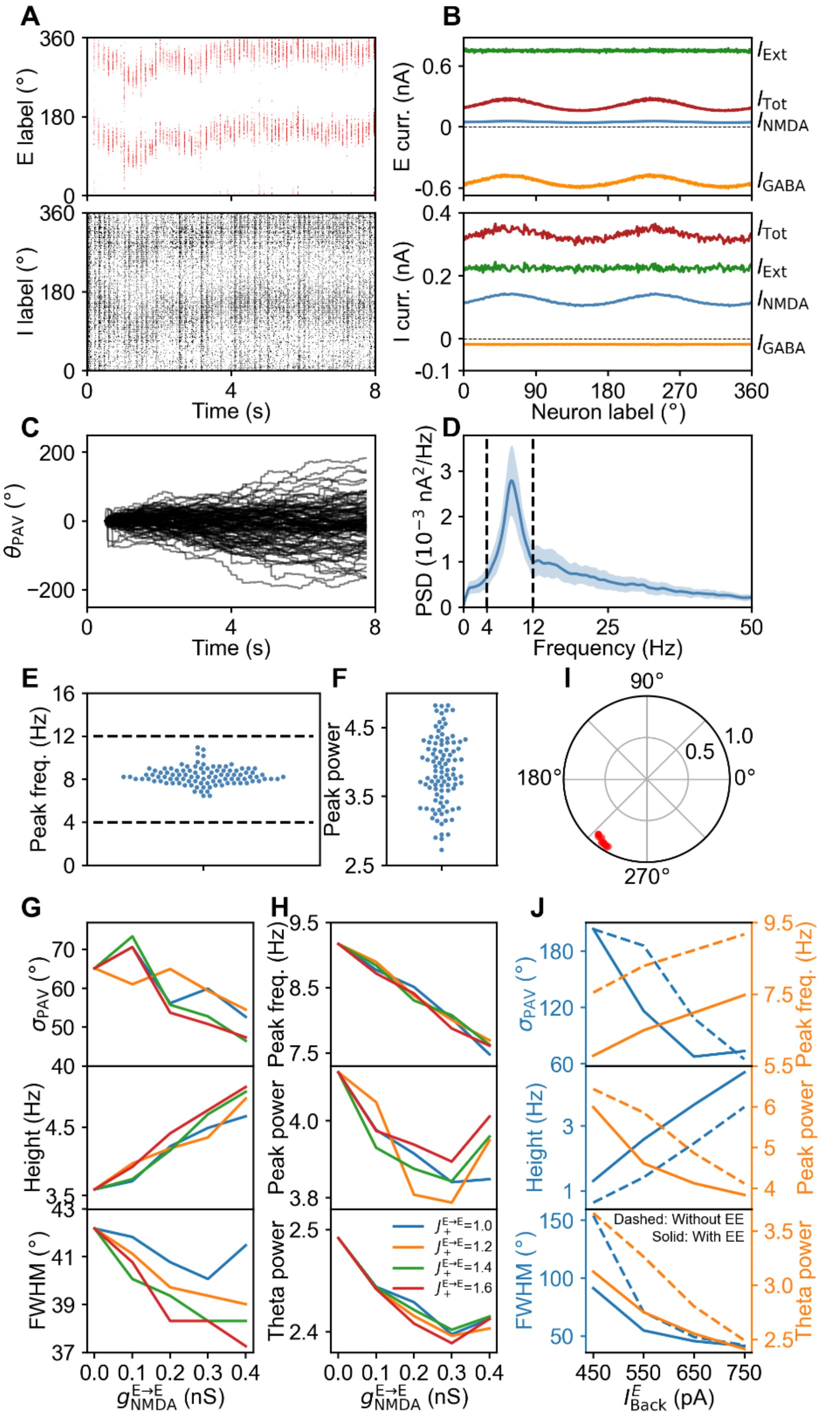

Figure S8. Network behavior in the presence of recurrent excitation between principal cells (related to Figure 5)

(A) Raster plots for principal cells (upper) and interneurons (lower). 1024 principal cells and 256 interneurons are labeled by their angular positions and arranged along y-axis in order. Red (black) dots on each row mark spikes of that principal cell

(interneuron). No brief current is applied here. $g_{\mathrm{NMDA}}^{\mathrm{E}\to\mathrm{E}}$ = 0.2 nS and $J_{+}^{\mathrm{E}\to\mathrm{E}}$ = 1.4 in (A-F).

(B) Sources of current input to principal cells (upper) and interneurons (lower). Note that I_{NMDA} to principal cells is greater than 0.

(C) Time courses of the angle of the population activity vector (θ_{PAV}) on 100 trials.

(D) Power spectral density (PSD) of pLFP averaged over 100 trials. The light shadow marks the standard deviation.

(E) Swarm plot of the primary peak frequency in the PSD of pLFP on 100 trials (8.30 ± 0.79 Hz).

(F) Swarm plot of the primary peak power in the PSD of pLFP on 100 trials (3.88 ± 0.497).

(G) Standard deviation of θ_{PAV} at 7 s (σ_{PAV}; upper), height (middle) and full width at half maximum (FWHM, lower) of bump attractors versus $g_{\mathrm{NMDA}}^{\mathrm{E}\to\mathrm{E}}$.

(H) Primary peak frequency (upper), peak power (middle) and theta power (lower) in the PSD of pLFP versus $g_{\mathrm{NMDA}}^{\mathrm{E}\to\mathrm{E}}$. Different colors label distinct values of $J_{+}^{\mathrm{E}\to\mathrm{E}}$ in (G) and (H).

(I) Phase-locking index $\boldsymbol{\Phi}$ of the 90° principal cell firing relative to the pLFP for 20 cases. Each data point is an average over 100 trials.

(J) Standard deviation of θ_{PAV} at 7 s (σ_{PAV}; blue, top), height (blue, middle) and full width at half maximum (FWHM; blue, bottom) of bump attractors, and the primary peak frequency (orange, top), peak power (orange, middle), and theta power (orange, bottom) in the PSD of pLFP versus $I_{\mathrm{Back}}^{\mathrm{E}}$ with (solid) or without (dashed) recurrent excitation between principal cells.

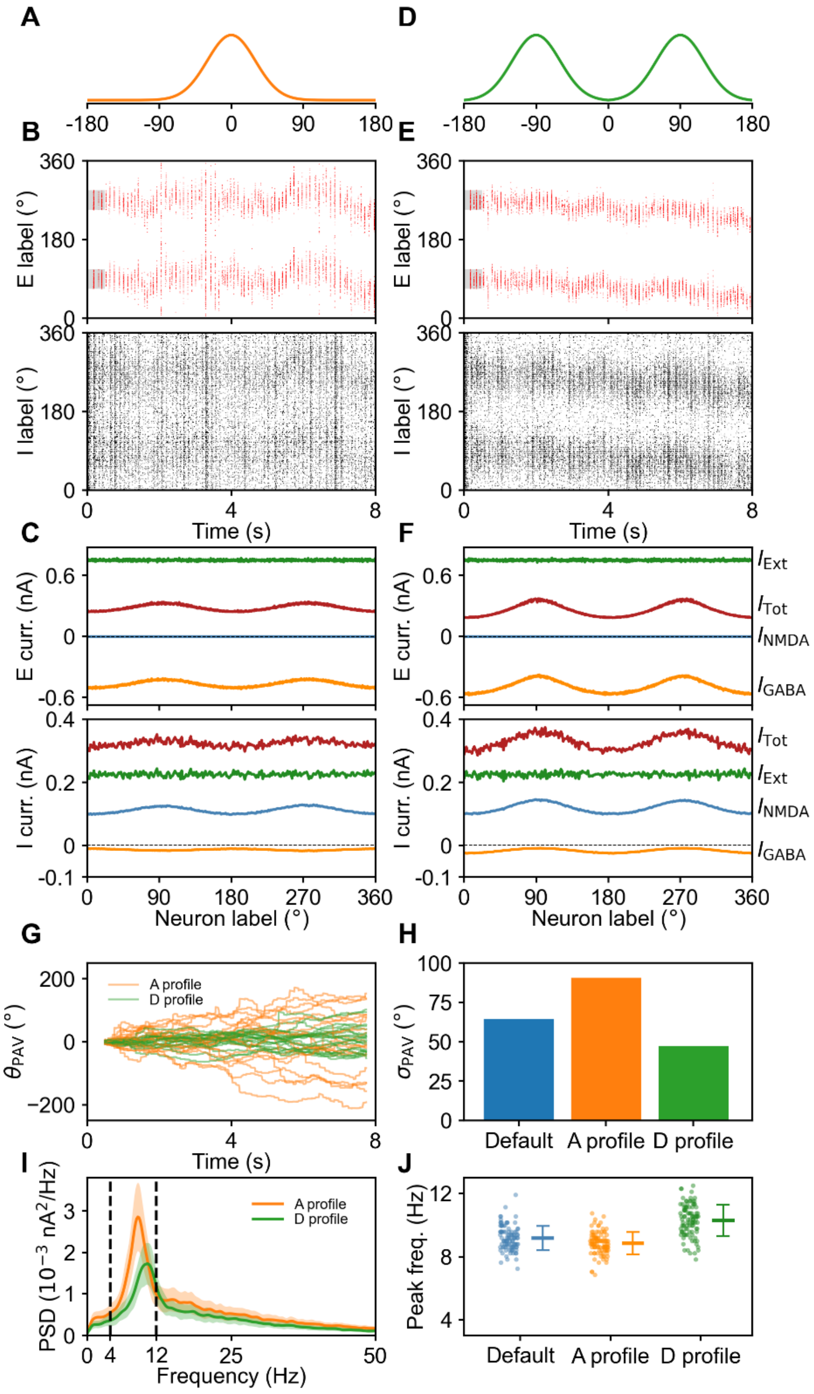

Figure S9. Network behavior with structured connectivity between interneurons (related to Figure 5)

(A-F) The left and right columns separately correspond to the case where the profile of connectivity strength between interneurons is a unimodal or bimodal function of their angular difference. (A/D) Schematic of the connectivity strength profile. (B/E) Raster plots for principal cells (upper) and interneurons (lower); 1024 principal cells

and 256 interneurons are labeled by their angular positions and arranged along y-axis in order. Red (black) dots on each row mark spikes of that principal cell (interneuron). The gray shadows indicate where and when a brief current is applied. (C/F) Sources of current input to each principal cell (upper) and interneuron (lower).

(G) Time courses of the angle of the population activity vector (θ_{PAV}) on 20 trials.

(H) Standard deviation of θ_{PAV} at 7 s for the default uniform connectivity and structured connectivity between interneurons.

(I) Power spectral density (PSD) of pLFP averaged over 100 trials in the cases of structured I-I connectivity. The light shadow marks the standard deviation.

(J) Primary peak frequency in the PSD of pLFP over 100 trials for the three cases: 9.17 ± 0.76, 8.84 ± 0.70 and 10.29 ± 0.99 Hz (from left to right). The bars at the right indicate the mean and standard deviation.

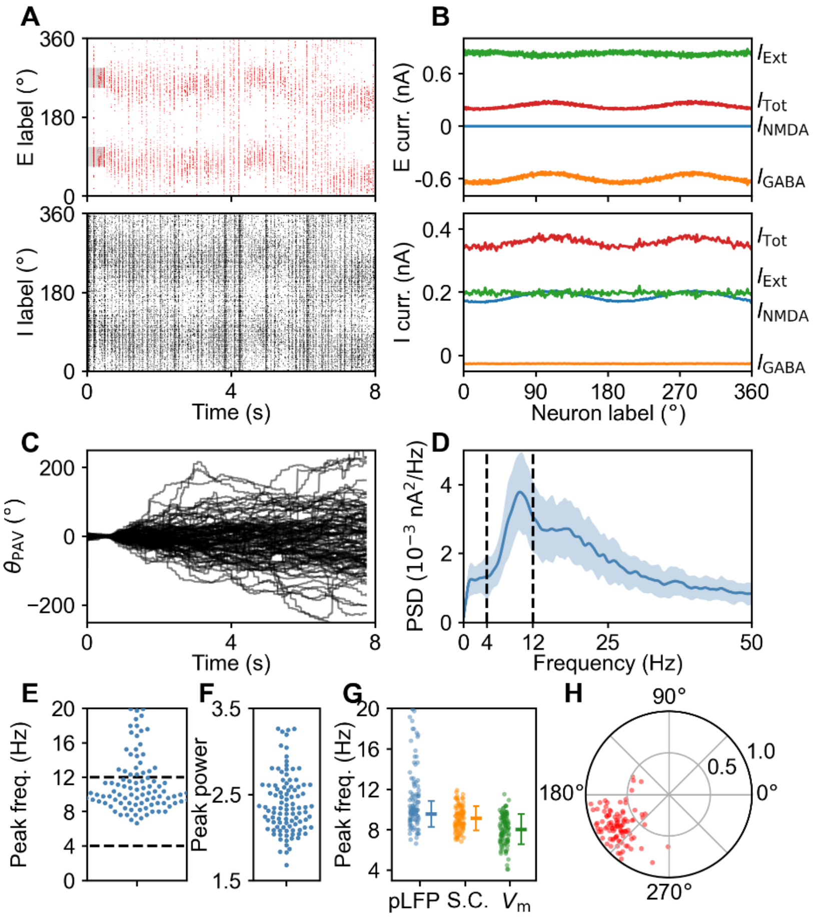

Figure S10. Network behavior when the background and MS inputs are modeled as synaptic currents from Poisson spike trains (related to Figure 6)

(A) Raster plots for principal cells (upper) and interneurons (lower). 1024 principal cells and 256 interneurons are labeled by their angular positions and arranged along y-axis in order. Red (black) dots on each row mark spikes of that principal cell (interneuron). The gray shadows indicate where and when a brief current is applied.

(B) Sources of current input to principal cells (upper) and interneurons (lower).

(C) Time courses of the angle of the population activity vector (θ_{PAV}) on 100 trials.

(D) Power spectral density (PSD) of pLFP averaged over 100 trials. The light shadow marks the standard deviation.

(E) Swarm plot of the primary peak frequency in the PSD of pLFP on 100 trials (9.56 ±1.28 Hz).

(F) Swarm plot of the primary peak power in the PSD of pLFP on 100 trials (2.39 ±

0.342).

(G) Strip plots summarizing the peak frequency for three quantities: the pLFP (9.56 ± 1.28 Hz), spike count from all principal cells in bins of 5 ms (S. C.; 9.15 ± 1.19 Hz), and membrane potential of the 90° principal cell (V_m; 8.04 ± 1.48 Hz).

(H) Phase-locking index $\boldsymbol{\Phi}$ of the 90° principal cell firing relative to the pLFP on 100 trials, with its mean arg($\boldsymbol{\Phi}$) and |$\boldsymbol{\Phi}$| equaling 211.4° and 0.708, respectively.

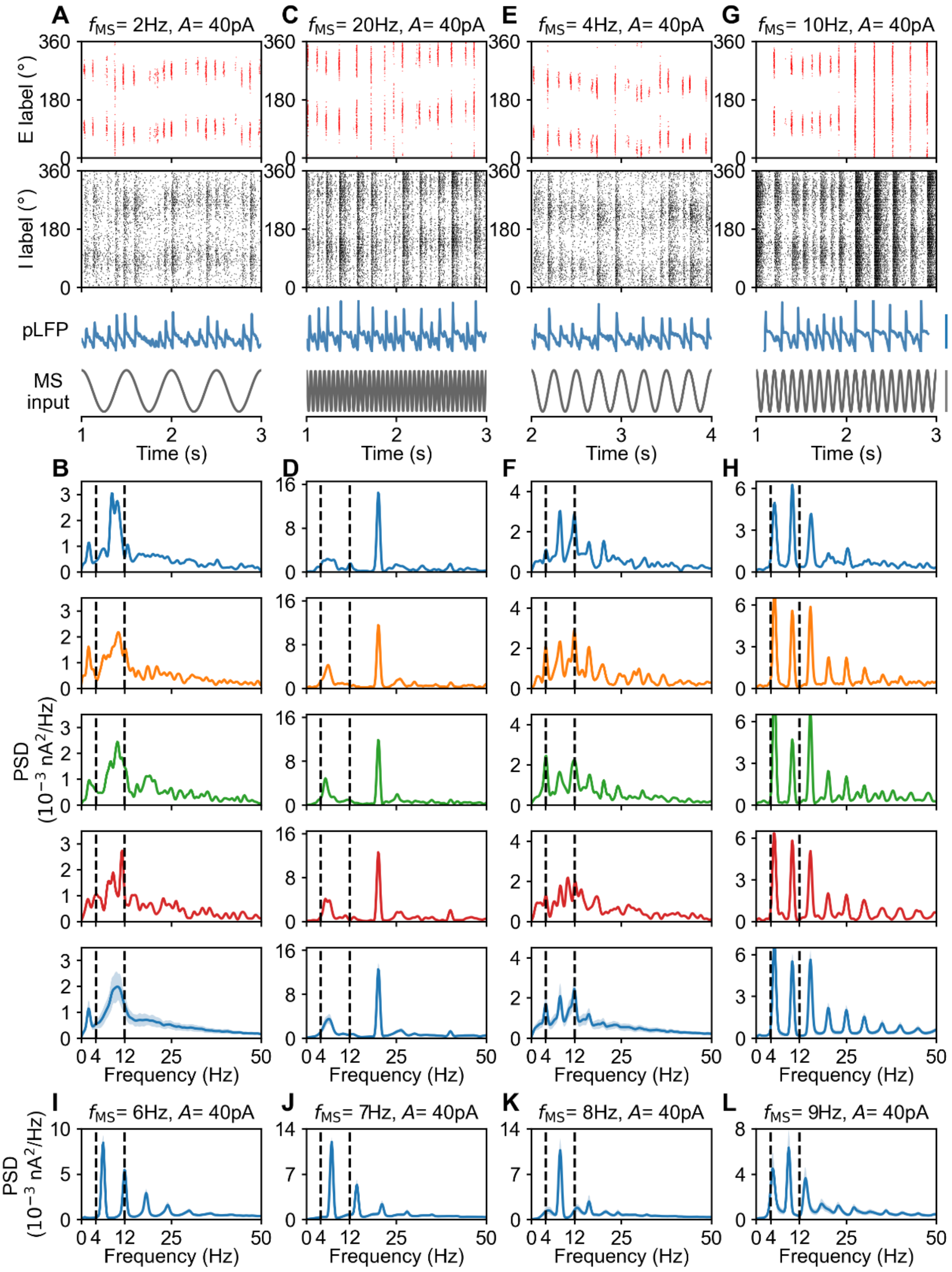

Figure S11. Examples of neural dynamics under various MS input rhythm at A = 40 pA (related to Figure 7)

The frequency f_{MS} of the MS input is 2 Hz (A, B), 20 Hz (C, D), 4 Hz (E, F), 10 Hz (G, H), 6 Hz (I), 7 Hz (J), 8 Hz (K), or 9 Hz (L). The region between two dashed lines in the PSD marks the theta range.

In (A, C, E, G), each row shows the raster plots for principal cells and interneurons, time courses of the pLFP and the MS input (from top to bottom). The bar at the right indicates 1 nA for pLFP or 80 pA for the MS input.

In (B, D, F, H), the top four rows show examples of the PSD of pLFP over 1-8 s (the first row shows the data from the simulation in (A, C, E, G)), while the bottom row displays the PSD of pLFP averaged over 100 trials.

In (I-L), shown is the PSD of pLFP averaged over 100 trials.

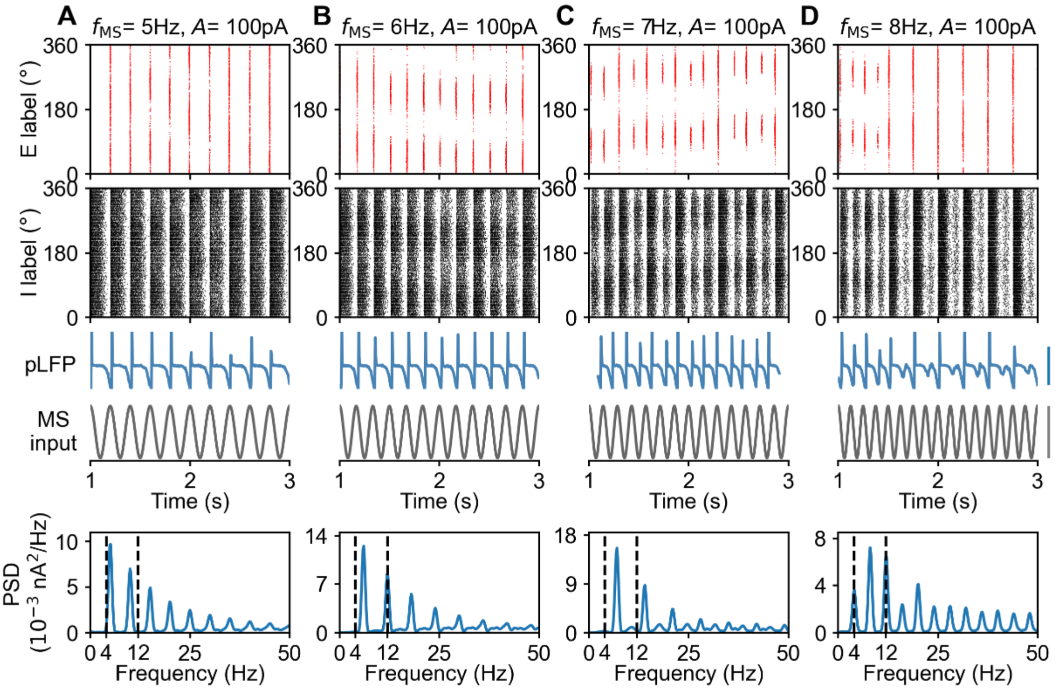

Figure S12. Examples of neural dynamics under various MS input rhythm at *A* = 100 pA (related to Figure 7)

The frequency f_{MS} of the MS input is 5 Hz (A), 6 Hz (B), 7 Hz (C), or 8 Hz (D). In each panel shown are the raster plots for principal cells and interneurons, time courses of the pLFP, MS input and PSD of the pLFP over 1-8 s (from top to bottom). The bar at the right indicates 1 nA for the pLFP or 200 pA for the MS input. The region between two dashed lines in the PSD marks the theta range.

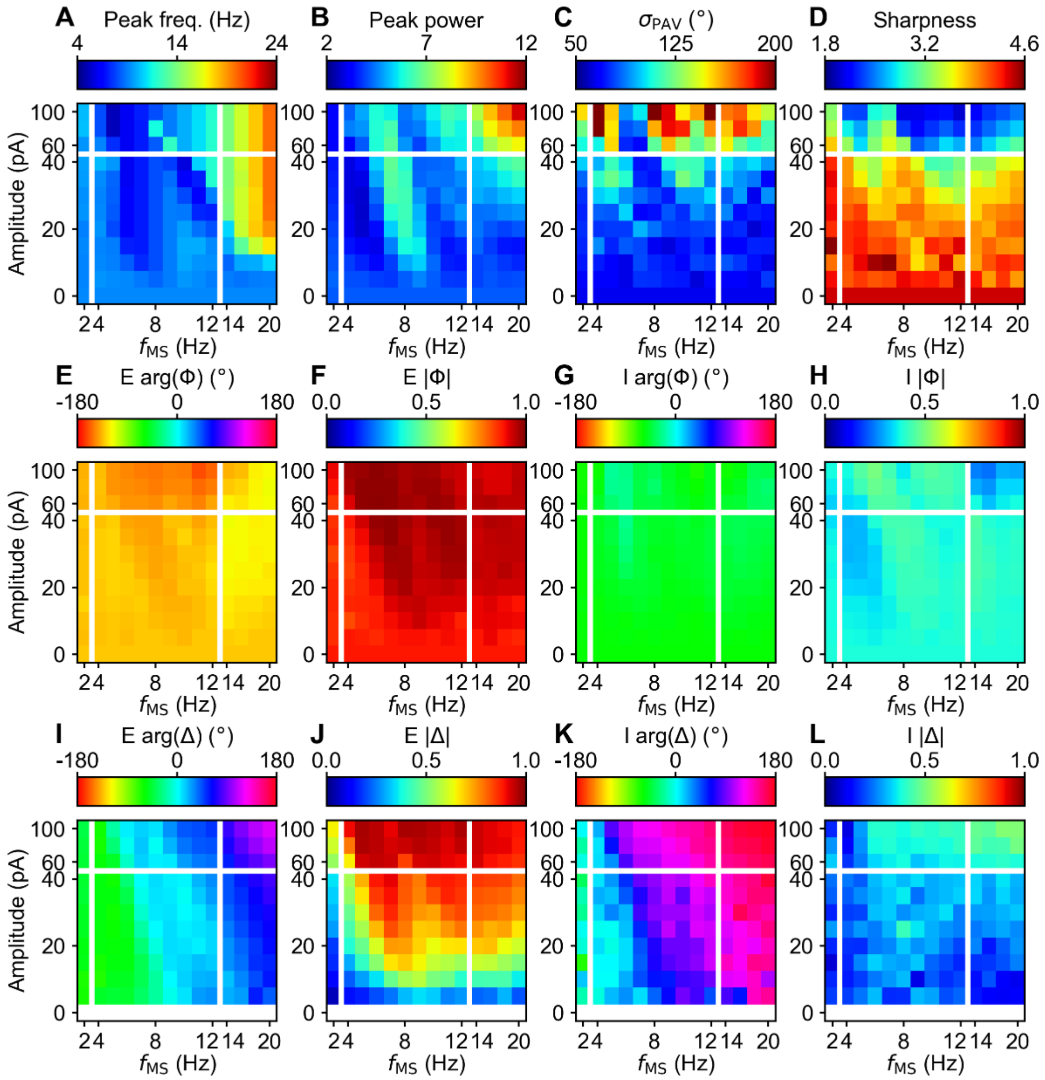

Figure S13. Dependence of the sustained and rhythmic activity on the amplitude and frequency of the MS input (related to Figure 7)

The value of each quantity is color coded as a function of the amplitude and frequency. For each parameter set, an average over 100 trials was taken. The white lines label the cases where parameter values are selected uniformly.

(A) Mean peak frequency in the PSD of the pLFP.

(B) Mean peak power in the PSD of the pLFP.

(C) Standard deviation of θ_{PAV} (σ_{PAV}) at 7 s.

(D) Mean sharpness of the bump attractors.

(E) and (F) arg($\boldsymbol{\Phi}$) and |$\boldsymbol{\Phi}$| of the principal cell at 90° firing relative to the pLFP theta rhythm.

(G) and (H) arg($\boldsymbol{\Phi}$) and $|\boldsymbol{\Phi}|$ of the interneuron at 90° firing relative to the pLFP theta rhythm.

(I) and (J) arg($\boldsymbol{\Delta}$) and $|\boldsymbol{\Delta}|$ of the principal cell at 90° firing relative to the MS input rhythm.

(K) and (L) arg($\boldsymbol{\Delta}$) and $|\boldsymbol{\Delta}|$ of the interneuron at 90° firing locked to the MS input rhythm.
